# Comparative mutant analyses reveal a novel mechanism of ARF regulation in land plants

**DOI:** 10.1038/s41477-025-01973-3

**Published:** 2025-04-11

**Authors:** Michael J. Prigge, Nicholas Morffy, Amber de Neve, Whitnie Szutu, María Jazmín Abraham-Juárez, Trisha McAllister, Heather Jones, Kjel Johnson, Nicole Do, Meirav Lavy, Sarah Hake, Lucia C. Strader, Mark Estelle, Annis E. Richardson

**Affiliations:** 1 Department of Cell and Developmental Biology, School of Biological Sciences, University of California San Diego, La Jolla, CA USA; 2 Department of Biology, Duke University, Durham, NC USA; 3 USDA Plant Gene Expression Center, Albany, CA USA; 4 Department of Plant and Microbial Biology, University of California Berkeley, Berkeley, CA USA; 5 Laboratorio Nacional de Genómica para la Biodiversidad (LANGEBIO), Unidad de Genómica Avanzada, Centro de Investigación y de Estudios Avanzados (CINVESTAV), Irapuato, Mexico; 6 Institute of Molecular Plant Sciences, School of Biological Sciences, University of Edinburgh, Edinburgh, UK

**Keywords:** Auxin, Plant development, Plant genetics, Plant molecular biology

## Abstract

The plant hormone auxin regulates a wide variety of transcriptional responses depending on the cell type, environment and species. How this diversity is achieved may be related to the specific complement of auxin-signalling components in each cell. The levels of activators (class-A AUXIN RESPONSE FACTORS) and repressors (class-B ARFs) are particularly important. Tight regulation of ARF protein levels is probably key in determining this balance. Through comparative analysis of novel, dominant mutants in maize and the moss *Physcomitrium patens*, we have discovered a ~500-million-year-old mechanism of class-B ARF protein-level regulation mediated by proteasome degradation, important in determining cell fate decisions across land plants. Thus, our results add a key piece to the puzzle of how auxin regulates plant development.

## Abstract (web summary)

Comparative analysis of maize and moss reveals a 500-million-year-old piece of the auxin-signalling puzzle that controls the protein level of ARFs, key transcriptional regulators.

## Main

Plants develop organs continuously through the activity of stem cells. Pattern formation, differentiation and tissue deformation define the shape of complex organs, while iterative development of these organs builds the plant body. Modulation of these developmental processes gives rise to a huge diversity in organ shapes and plant architectures. Despite this complexity, a single phytohormone, auxin, has a major conserved role across land plants^1–4^, underpinning cell fate decisions, organ initiation, shape and plant architecture. How auxin precisely regulates so many distinct processes is one of the biggest unresolved questions in plant biology.

Canonical auxin signalling occurs through the activity of a suite of deeply conserved transcriptional regulators found in all land plants^3,5–7^. The AUXIN/INDOLE-3-ACETIC-ACID repressors (AUX/IAAs), AUXIN RESPONSE FACTORS (ARFs) and the TRANSPORT INHIBITOR RESPONSE 1/AUXIN-SIGNALING F-BOX (TIR1/AFB) co-receptors are components of the best-characterized auxin-mediated transcriptional regulation pathway^8–10^. All three components are in multigene families that have greatly expanded in flowering plants^3^. When auxin levels are low, AUX/IAAs are bound to ARFs and recruit transcriptional co-repressors, preventing ARF activation of auxin-responsive genes. In elevated auxin levels, the TIR1/AFB co-receptor forms a complex with auxin and AUX/IAA repressors, resulting in ubiquitylation and degradation of AUX/IAAs via the 26S proteasome^11–13^. Degradation of AUX/IAAs relieves ARF repression, facilitating auxin transcriptional responses^14^. Recent studies have elaborated on this core pathway, showing that the TIR1/AFB proteins possess adenyl cyclase activity activated upon auxin binding and required for ARF activity^15^. In addition, the ABP1-TMK pathway, responsible for perceiving extracellular auxin, can influence ARF function by phosphorylating the non-canonical Aux/IAA proteins IAA32 and IAA34 (ref. ^16^).

Land plant ARFs are divided into three phylogenetic lineages; class-A, -B and -C, each associated with functions based on protoplast and *Marchantia* studies. Class-A ARFs are activators, while class-B and -C are potential repressors^4,17,18^. All ARFs share a DNA-binding domain (DBD), a middle domain and a C-terminal Phox and Bem1 (PB1) domain^19,20^. Differences in domain sequences lead to variation in DNA-binding activity^21–25^, interacting partners (including other ARFs, AUX/IAAs, other transcription factors, chromatin remodellers^19^) and oligomerization^26^. Modulation of auxin response is believed to be determined by the complement of ARF and AUX/IAA proteins in the cell, and in vitro analysis has revealed different activation levels of ARF–AUX/IAA protein combinations, indicating potential subfunctionalization mechanisms^27^. However, the elegant model of auxin-dependent ARF de-repression is not able to account for all auxin responses. For example, only 5 of the 22 *Arabidopsis thaliana* (Arabidopsis hereafter) ARFs functionally interact with AUX/IAAs in vivo^19^.

Although genetic studies implicate class-B function in diverse developmental processes^28–30^, their mode of action and regulation is unclear. Studies in non-vascular plants such as *Marchantia* and *Physcomitrium* indicate that class-B ARFs can directly compete with class-A ARFs to bind to promoter sequences^13,31^. In maize, class-A and -B ARFs exhibit clear DNA-binding site differences. However, a proportion of the sites (16%) are bound by both classes, thus supporting the hypothesis that competition between class-A and -B ARFs may be important for the regulation of some auxin-regulated genes across land plants^24,32,33^. In addition, class-B ARFs can function independently of AUX/IAAs, directly recruiting the TOPLESS (TPL) repressor to chromatin^34–38^, providing an AUX/IAA-independent way to repress gene expression. Regardless of mechanism, the level of class-B ARFs is a key determinant of auxin-regulated transcription. Without a clear understanding of class-B ARF protein regulation and how it has evolved, we lack a key piece of the auxin regulatory system puzzle.

To address this deficiency, we leveraged a comparative approach by investigating dominant class-B ARF mutants in the distantly related species *Zea mays* (maize) and the moss *Physcomitrium patens* (*P. patens*), which diverged ~500 million years ago (Ma)^39^. The mutations result in amino acid substitutions in a loop region in the DBD. We demonstrate that this contains a novel regulatory region that is required for degradation of class-B ARF proteins, thus revealing a deeply conserved mechanism of class-B ARF regulation in land plants.

## *Truffula* has pleiotropic defects

**Fig. 1:**
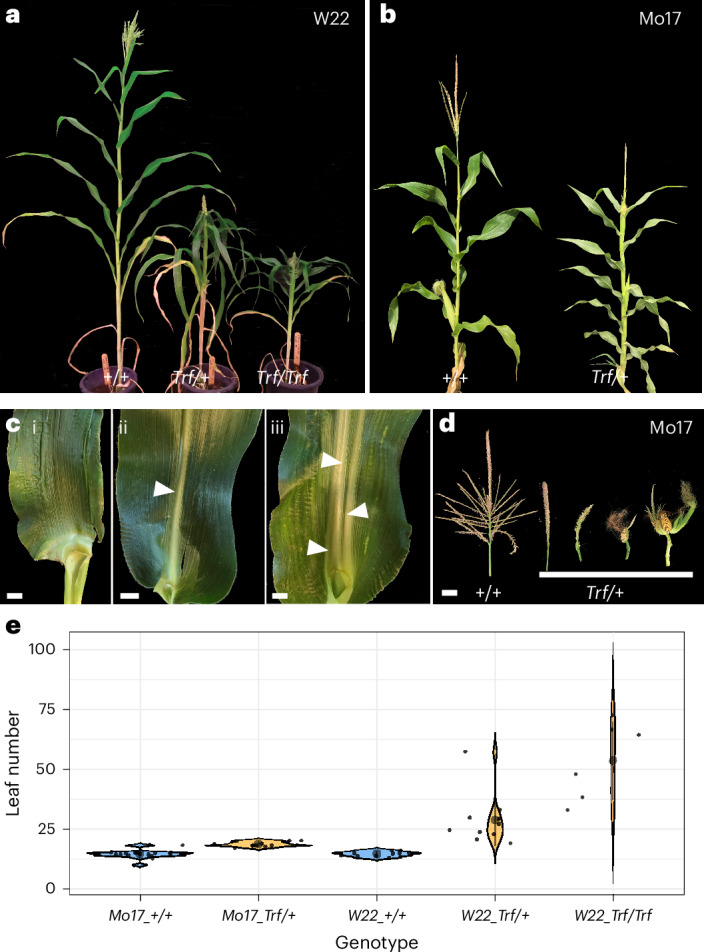
Maize *Truffula* mutants have pleiotropic defects. **a**–**d**, Normal (*+/+*) *and Trf* siblings (*Trf/+*, *Trf/Trf*) in the W22 (**a**,**c**) and Mo17 (**b**,**d**) inbred backgrounds. **a**,**b**, Mature plants. **c**, Midrib phenotypes in *Trf* leaves: midribless (**i**), normal (**ii**) and multiple midribs (**iii**). Scale bars, 1 cm. **d**, Tassel phenotypes showing ear-like traits in *Trf* siblings. Scale bar, 5 cm. **e**, Leaf number in mature plants of normal (blue) and *Trf* siblings (orange). **P < 0.05 determined by Kruskal–Wallis non-parametric test followed by Wilcox pairwise tests, *n* ≥ 6 plants. Black dot and error bars, mean ± s.d.

Maize is a classic model genetic system with a rich history of developmental mutant analyses^40^. We found the striking, dominant *Truffula* (*Trf*) mutant in an ethyl methanesulfonate (EMS)-mutagenized population and analysed it in two inbred backgrounds: W22 and Mo17. In both inbreds, when compared to normal siblings, *Trf* mutants had reduced plant height, increased leaf number, and tassels and tassel flowers with ear traits (Fig. 1a–d and Supplementary Fig. 1). The mutation did not transmit through the ovule parent in Mo17, but did at a reduced rate in W22, permitting analysis of homozygotes. Mutant plants were half the height of normal siblings in W22 (Fig. 1a) and two-thirds the size in Mo17 (Fig. 1b). The reduced height results from shorter internodes, the stem section between leaves (Supplementary Fig. 1a,b). On average in Mo17, non-mutant siblings produced 15 leaves, while heterozygous mutants produced 19. In W22, non-mutants produced 15 leaves, heterozygotes produced 29 and homozygotes produced 54 (Fig. 1e). Mutants flowered at the same time as normal siblings (Supplementary Fig. 1c), hence the increase in leaf number is due to a shorter plastochron (time between leaf initiations) in *Trf*. Leaf phyllotaxy was also altered in the upper nodes of *Trf*, switching from distichous to spiral-like (Supplementary Fig. 1d). Additional *Trf* phenotypes were inbred dependent such as defects in midrib formation (15% of leaves), ranging from midribless leaves to multiple midribs (Fig. 1c, arrowheads), and leaf shape, forming very rare tube leaves in W22 (<1% of leaves). In W22, only a few kernels formed at the base of the normally staminate tassel, while in Mo17 many tassel florets were pistillate and tassel branches were often transformed into ears wrapped in husk leaves (Fig. 1d).

## *Trf* is associated with a mutation in *ZmARF28*

**Fig. 2:**
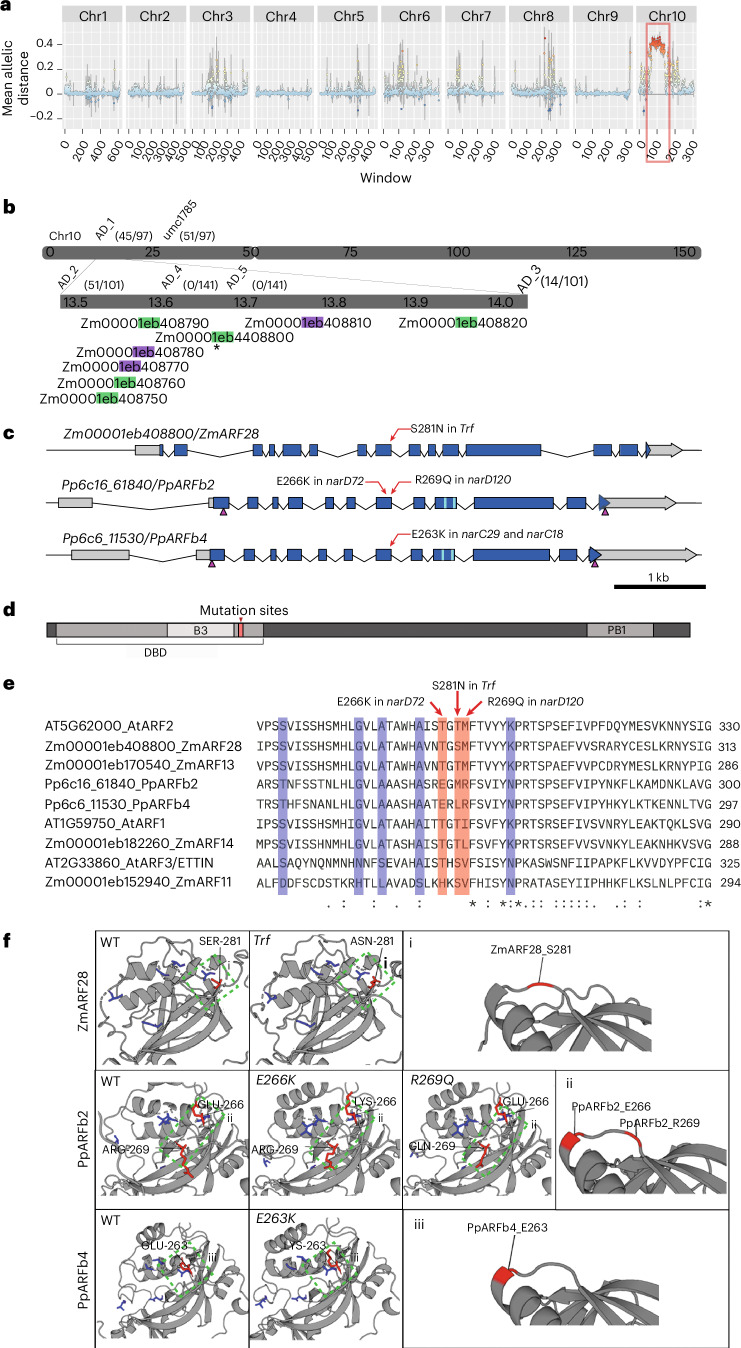
The *Truffula* lesion maps to *ZmARF28.* **a**, Plotting WGS-BSA variant mean allelic distances in 0.5-Mbp windows across the maize NAM5.0 reference genome reveals a region on chromosome 10 (Chr10) which is more associated with *Trf* mutants than normal siblings (red box). *n* = 80 per genotype. Dot and error bars, mean ± s.e.m. Colour scale: mean allelic distance −0.25:dark blue to 0.45:red. **b**, PCR mapping narrows the region to between 13.5 Mbp and 14.0 Mbp. Mapping primer positions are indicated (AD_# and umc#), numbers represent Mbp position on Chr10, and recombinant numbers are shown in brackets. There are 8 genes in the interval, of which 5 have an FPKM > 1 in our shoot apical meristem RNA-seq dataset (green) and 3 have an FPKM < 1 (purple). A moderate effect, *Trf*-specific, EMS-type SNP (*) is present in Zm00001eb408800 (*ZmARF28*). **c**, Locations of the *Trf, narD72, narD120, narC29* and *narC18* mutations (red arrows). The exons (blue), 5’ and 3’ UTRs (grey), CRISPR sgRNA targets for deletions (magenta arrowheads), and small RNA regulatory sites (cyan) are indicated. **d**, Generalized cartoon depiction of the protein domains in class-B ARFs, with the DBD, B3 and PB1 domains indicated; the relative location of the mutations identified in this study are indicated in red. **e**,**f**, The maize *Trf* mutation and the moss *narD72, narD120, narC29* and *narC18* mutations map to the same loop region in the ARF protein. **e**, Multiple sequence alignments of ARFs from maize, Arabidopsis and moss. **f**, Homology modelling of the ZmARF28, PpARFb2 and PpARFb4 DBDs for both wild-type and mutant proteins (metrics in Supplementary Table 10). (**i**)–(**iii**), Zoomed-in images of the green-boxed regions. **e**,**f**, Mutation locations (red) and amino acids previously shown to be important for ARF function (blue) are indicated.

To identify the underlying causal mutation in *Trf*, we carried out whole-genome sequencing (WGS) bulk-segregant analysis^41^, identifying a region between 11.0 Mbp and 20.0 Mbp on chromosome 10 (Fig. 2a). Short sequence repeat (SSR) mapping (Fig. 2b and Supplementary Table 1) refined the region to between 13.5 Mbp and 14.0 Mbp. This region contains 8 annotated genes, of which 5 are expressed in the vegetative shoot apex according to RNA-seq (Fig. 2b, and Supplementary Fig. 2a and Table 2). Analysis of the WGS data in the mapping region reveals 188 EMS-type variants (G:C to A:T transitions) specific to *Trf* siblings. Of these SNPs, only one is in a conserved non-coding sequence, associated with Zm00001eb408820, a gene which is not significantly differentially expressed in *Trf*. SNPEff^42^ analysis predicts 7 moderate to high-effect EMS-type single nucleotide polymorphisms (SNPs) in the interval (Supplementary Table 3). Only a single SNP in exon 8 of ZmAUXIN RESPONSE FACTOR28 *(ZmARF28*, Zm00001eb408800), resulting in a Ser-to-Asn change in the DBD (Fig. 2c–f), is *Trf* specific when compared to other maize inbred backgrounds (Supplementary Table 3), suggesting that this mutation in *ZmARF28* is probably responsible for the *Trf* phenotype.

The *Trf* phenotype, although distinct from other reported *ARF* mutants in maize^43^, is consistent with the defects observed in maize seedlings treated with auxin transport inhibitors^44^ and other auxin-signalling mutants^43^ in which changes to organ initiation and medial-lateral leaf patterning are observed. To investigate the link between *Trf* and auxin response further, we carried out an RNA-seq analysis of vegetative shoot apices, at a time consistent with the formation of the *Trf* vegetative phenotypes (Supplementary Fig. 2a–d and Table 4). A total of 1,804 genes were significantly differentially expressed (sigDE), of which 455 were downregulated (*P*_adj_ < 0.05, log_2_fold change (FC) < −0.5). Interestingly, when compared to published class-B ARF DNA affinity purification sequencing (DAP-seq) data^24^, potential class-B ARF target genes were both up- and downregulated (19% and 16%, respectively, Supplementary Fig. 2d), suggesting that ZmARF28’s role may not be only as a straightforward repressor. Consistent with a defect in an ARF, sigDE genes were enriched for Gene Ontology (GO) terms associated with auxin signalling and response (*P*_adj_ = 0.00002, Supplementary Table 5). In line with the *Trf* phenotype, we also observed enrichments for GO terms associated with meristem development (Supplementary Fig. 2c). Crucially, this RNA-seq data showed that ZmARF28 is not significantly differentially expressed in *Trf* (Supplementary Fig. 2b), suggesting that the *Trf* mutation affects protein activity or post-translational regulation of ZmARF28. To further evaluate the auxin response in *Trf*, we treated seedlings with indole-acetic-acid (IAA) and evaluated transcriptional response using RNA-seq and quantitative (q)PCR (Supplementary Fig. 2e–h and Tables 6 and 7). At 30 min post-IAA treatment, both normal and *Trf* siblings showed significant differential gene expression in response to auxin, although the overlap is small (Supplementary Fig. 2f), suggesting differential responses potentially arising from the existing dysregulation of auxin-signalling components in *Trf* seedlings before treatment. Of the 1,130 sigDE genes, 831 genes were downregulated in *Trf* siblings treated with IAA versus mock (*P*_adj_ < 0.05, log_2_FC < −0.5), consistent with a role as a repressor of auxin-responsive gene expression. Of the 299 significantly upregulated genes (*P*_adj_ < 0.05, log_2_FC > 0.5), 58 were potential class-B ARF targets based on overlap with published DAP-seq data^24^ (Supplementary Fig. 2g), and 5 of these were AUX/IAA genes, highlighting a potential role of ZmARF28 in promoting the expression of other repressors of auxin response. Mapping fragments per kilobase of transcript per million mapped reads (FPKM) of all genes associated with auxin GO terms and expressed in both *Trf* and normal siblings showed clear auxin transcriptional response differences (Supplementary Fig. 2h). Combined, this evidence supports the hypothesis that the *Trf* phenotype is at least in part caused by altered auxin transcriptional response due to the identified mutation in *ZmARF28*.

As *ZmARF28* is a co-orthologue of Arabidopsis *AtARF2* (Supplementary Fig. 3), we carried out sequence alignments (Fig. 2e) to see whether the mutation was in an already characterized residue. We found that although the mutation is in the DBD, the *Trf* mutation is in the dimerization domain, not the B3 domain involved in DNA binding, and is outside of the core residues previously shown to be required for functionality in AtARF1 and AtARF5 (Fig. 2d–f, blue, ref. ^21^). Homology modelling revealed that the *Trf* residue is in an externally facing loop region and is not predicted to cause notable structural changes (Fig. 2f). These findings suggest that the novel *Trf* phenotype is not due to a mutation in an already characterized ARF residue, potentially revealing a novel regulatory module.

Although protein structure is predicted to be conserved, amino acid sequence is variable in this region of plant class-B ARFs (Fig. 2e). This suggests that the *Trf* phenotype could be due to the disruption of a grass-specific regulatory pathway in the expanded family of class-B ARFs. Alternatively, the *Trf* mutation could highlight the role of a functionally conserved region of class-B ARFs that has yet to be described due to lack of sequence conservation.

## Mutations in *P. patens* class-B ARFs cause auxin resistance

**Fig. 3:**
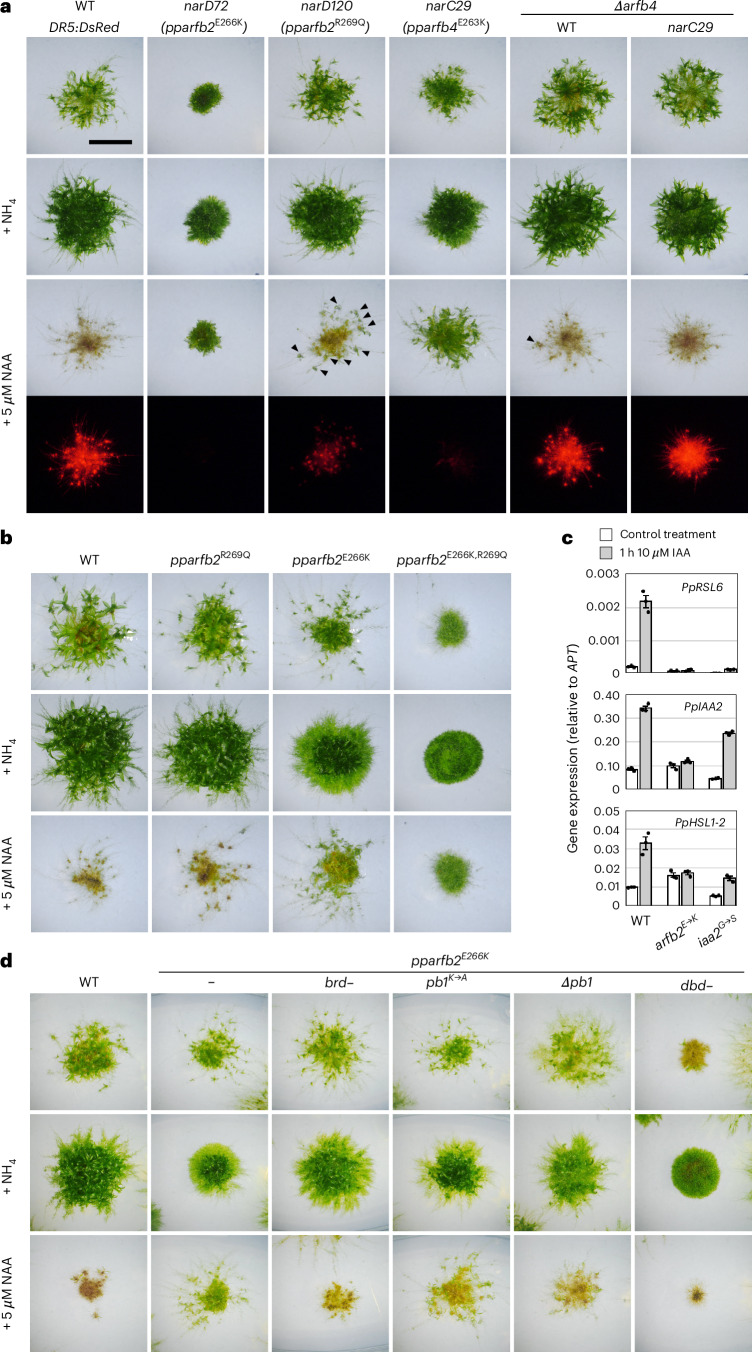
Mutations in *PpARFb2* and *PpARFb4* cause auxin resistance. **a**, Comparison of wild type, *nar* and *∆pparfb4* deletion mutants after 21 days of growth on standard medium (BCD) and BCD supplemented with 5 mM ammonium tartrate or 5 µM NAA. Plants grown on NAA were imaged for RFP fluorescence from the *proDR5:DsRed* auxin-response reporter. On NAA-containing media, wild-type and *∆pparfb4* strains produce ectopic rhizoids and high levels of RFP fluorescence, whereas the *nar* mutants produce green leafy gametophores. Arrowheads indicate leafy shoots in *narD120* and *∆pparfb4*. Scale bar, 5 mm. **b**, Comparison of wild type and gene-edited point mutants grown for 21 days, as in **a**. **c**, Expression of select auxin-responsive genes (*PpRSL6, ROOT HAIR DEFECTIVE SIX-LIKE 6*; *PpIAA2, Aux/IAA2*; *PpHSL1-2*, *HOOKLESS1*-*LIKE 2*) in wild type, *pparfb2*^*E266K*^ and auxin-response mutant *ppiaa2*^*G325S*^ grown for 1 h on media containing 10 µM IAA or 0.01% ethanol (3 technical replicates). Bars represent mean ± s.e.m. Transcript levels were normalized to the *APT* gene. **d**, Growth comparisons of wild type, the *pparfb2*^*E266K*^ mutant and the *pparfb2*^*E266K*^ mutant with second-site mutations, as in **a**. *brd–*, L614A + F615A substitutions in the B3 repression domain; *pb1K*→*A*, K682A substitution in the PB1 domain; *∆pb1*, a 5-bp frameshift-causing deletion starting at G680 codon; *dbd–*, P194A + R196A substitutions affecting DNA-interacting residues. *n* ≥ 4. Scale bars, 5 mm.

Evidence that the *Trf* mutation revealed an ancient mechanism of class-B ARF regulation emerged from parallel forward-genetic analyses in the moss *P. patens*. We found that the genomes of four newly isolated *P. patens NAA-RESISTANT (nar)* mutants^45^ had missense mutations in the same *Trf* region in two of the four class-B ARF genes (*PpARFb2* and *PpARFb4*, Fig. 2c–f). *narC29* and *narC18* mutants have missense mutations, causing Glu-to-Lys substitutions (E263K) in PpARFb4/Pp6c6_11530, while *narD72* has the same substitution in PpARFb2/Pp6c16_6840 (E266K). *narD120* has a Gln-to-Arg substitution in PpARFb2 (R269Q). When the DBD are aligned, the residues affected in the moss mutants flank the position altered by the maize *Trf* mutation (Fig. 2e,f). These class-B ARF moss *nar* mutants had clear defects in the auxin-regulated transition from highly photosynthetic chloronemal cells to ‘nutrient foraging’ spreading caulonemal cells, and a subsequent delay in the production of 3D-leafy shoot (gametophore) growth forms. This effect was more pronounced on media containing ammonium which delays transitions promoted by auxin^46^. Unlike wild type, the *nar* mutants produce gametophores even when grown on media containing micromolar amounts of 1-naphthaleneacetic acid (NAA) (Fig. 3a). Further supporting the reduction in auxin response in these *nar* mutants, expression of the *proDR5:DsRed2* auxin-signalling reporter was also dramatically reduced in *narC29*, *narC18, narD72* and to a lesser extent in *narD120* (Fig. 3a and Supplementary Fig. 4a).

To independently verify that *pparfb* mutations are responsible for the above *nar* phenotypes, we used oligo-mediated gene editing^47^ to introduce the E-to-K substitution to all four *PpARFb* loci. The *pparfb2*^*E266K*^ and *pparfb4*^*E263K*^ lines recapitulated the *nar* phenotypes of *narD72* and *narC29*, respectively, and the *pparfb1*^*E266K*^ and *pparfb3*^*E266K*^ lines were also appreciably resistant to NAA (Fig. 3b and Supplementary Fig. 4b). *pparfb2*^*R269Q*^ and *pparfb4*^*R266Q*^ mutations conferred weak *nar* phenotypes similar to *narD120* (Fig. 3b and Supplementary Fig. 4c). Interestingly, combining both the E-to-K and R-to-Q mutations in the same gene resulted in dramatically stronger phenotypes approaching those of quadruple mutants lacking all four *TIR1*/*AFB* genes which are completely auxin insensitive (Fig. 3b and Supplementary Fig. 4c). In-frame deletions and insertions in this region of *PpARFb2* also conferred either intermediate phenotypes similar to those of *pparfb2*^*E266K*^ or strong phenotypes similar to those of *pparfb2*^*E266K+R269Q*^ (Supplementary Table 8). Reduced expression of *proDR5:DsRed* and reduced auxin induction of auxin-regulated genes in *pparfb2*^*E266K*^ confirmed that the phenotypes are due to reduced auxin response (Fig. 3c and Supplementary Fig. 4d–e). Interestingly, *Trf*-like substitutions in *PpARFb2* (M268N) and *PpARFb4* (L265N) caused no phenotypic changes, but both *Trf*-like and *ZmARF28*-like 9-amino-acid swaps in the loop caused *nar* phenotypes (Supplementary Fig. 5).

The phenotypes of the four *nar* mutants are similar to those of lines that over-accumulate PpARFb protein either by expression with a strong heterologous promoter^13^ or by disruption of the *miR1219* and *tasiARF* target sequences^46^ (Supplementary Fig. 4f). The *nar* mutations do not affect any known small RNA regulatory elements, but the phenotypic similarities suggest that the *nar* lesions represent dominant mutations. To test this, we used CRISPR/Cas9 to delete the mutated genes in the mutant lines. Deletion of either the wild-type *PpARFb2* or *PpARFb4* genes in moss has minor phenotypic effects^13^, but deletion of the mutated genes in the *nar* mutants restored NAA sensitivity indicating that, similar to the maize *Trf* mutant, the *P. patens pparfb2* and *pparfb4* variants are dominant mutations (Fig. 3a and Supplementary Fig. 4a,g) with defects in auxin signalling.

## All major domains are required for the dominant phenotype

ARF domains confer specific functionality: the B3 repression domain (BRD) recruits the TPL repressor^34,48^, the PB1 domain mediates oligomerization^49^, and the DBD binds target DNA sequences and can facilitate binding to other proteins^20,21^. To better understand how the above mutations confer gain-of-function auxin phenotypes in moss, we introduced second-site mutations affecting key residues of the known functional domains into the *pparfb2*^*E266K*^ mutant (Fig. 3d and Supplementary Fig. 6). We anticipated that if the functionality of a domain was required for the *pparfb2*^*E266K*^ phenotype, we would restore the wild-type phenotype. Mutating the BRD (L614S + F615S) eliminates the interaction with a TPL homologue (TPL2/Pp6c9_10690) and reverts the *pparfb2*^*E266K*^ phenotype to wild type (Fig. 3d and Supplementary Fig. 6a–c). Similarly, mutating a conserved lysine residue in the positive face of the PB1 domain (K682A) partially suppresses the *arfb2*^*E266K*^ phenotype, and a frameshift mutation near the start of the domain results in near-complete suppression. Introducing a pair of substitutions (P194A + R196A) shown to abolish DNA binding^21^ restores NAA response, although additional phenotypes appear, suggesting dominant-negative effects. None of these functional-domain mutations caused phenotypic effects in an otherwise wild-type *PpARFb2* (Supplementary Fig. 6a). These data show that the gain-of-function phenotypes depend on all known ARF protein functions: DNA binding, TPL recruitment and PB1-mediated oligomerization. Interestingly, the requirements of both the BRD and PB1 domains indicate that TPL recruitment through PB1 interactions with AUX/IAAs is insufficient and that oligomerization with other ARFs is required. Indeed, PpARFb4 overexpression phenotypes were previously shown to be independent of AUX/IAAs^13^.

## Mutations increase class-B ARF stability

**Fig. 4:**
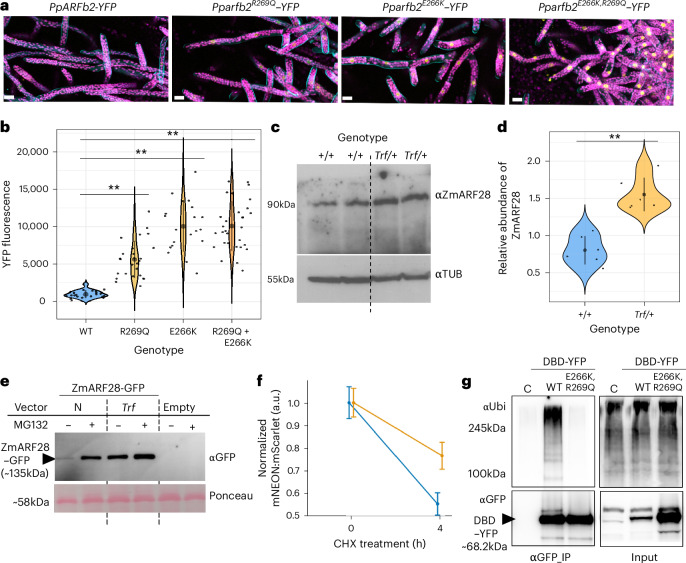
Mutations in the same domain result in stabilization of ZmARF28, PpARFb and AtARF2. **a**,**b**, PpARFb2-YFP signal in *P. patens* wild type, *pparfb2*^*R269Q*^, *pparfb2*^*E266K*^ and the *pparfb2*^*E226K,R269Q*^ double mutants. **a**, Representative confocal microscopy images. Yellow, PpARFb2-YFP; magenta, chlorophyll autofluorescence. Scale bar, 25 µm. **b**, Nuclear fluorescence quantification. Centre black dot and error bars, mean ± s.d.; **P < 0.01, determined using Kruskal–Wallis non-parametric test followed by Wilcox pairwise tests. **c**, Western blot of ZmARF28 in *+/+* and *Trf/+* mutant siblings from a W22 backcross population. Lanes are different biological replicates. Anti-tubulin was used as a loading control. **d**, Quantification of ZmARF28 abundance relative to TUBULIN. **P < 0.01, Welch 2-sample *t*-test. Centre black dot and error bars, mean ± s.d.; 3 technical replicates, 2 biological replicates in each. **e**, Western blots of ZmARF28-GFP accumulation in *N. bethamiana*, with (+) and without (−) MG132 treatment, *n* = 3. **f**, Mean ± s.e.m. ratiometric signal of AtARF2-mNeonGreen (blue) and AtARF2-Trf-mNeonGreen (orange) in Arabidopsis protoplasts, relative to mScarlet after cycloheximide (CHX) treatment (≥2,166 cells across 6 technical replicates analysed). **g**, Western blots of anti-GFP immunoprecipitated samples (αGFP IP) and cleared extracts (input) using anti-GFP and anti-ubiquitin antibodies. Extracts were from bortezomib-treated untransformed control (‘C’) and stable lines expressing YFP-fused ARFb2’s DBD with and without the E266K + R269Q substitutions. Mutant IP samples were diluted 5-fold to normalize DBD-YFP amounts loaded. *n* = 3. Source data

On the basis of the phenotypes and lack of *ZmARF28* and *PpARFb* expression changes in the mutants, we hypothesized that the maize *Trf* and *P. patens arfb* mutant phenotypes were due to accumulation of ZmARF28 and PpARFb protein, respectively. To test whether the PpARFb proteins are more abundant in the moss mutants, we knocked in mYPet yellow fluorescent protein (YFP) tags to the four endogenous *PpARFb* loci and the mutants to quantify protein abundance. The YFP signal dramatically increased in all nuclei of the mutants compared with the weak and sporadic YFP signal detected in nuclei in the wild type (Fig. 4a,b and Supplementary Fig. 7a,b). Protein localization did not change in the mutants (Fig. 4a). Since the transcript levels are similar in the mutant and wild-type lines (Supplementary Fig. 7c), the increased YFP signal suggests that the mutations affect PpARFb stability. Consistent with accumulation of PpARFb protein resulting in the mutant phenotypes, mutations with higher YFP signal had stronger mutant phenotypes and less auxin response (Fig. 3a, and Supplementary Figs. 4 and 5).

To determine whether the wild-type moss proteins are subject to degradation via the 26S proteasome, we treated the wild-type YFP lines overnight with the proteasome inhibitor, bortezomib^50^. Quantification of the YFP signal revealed significant increases in protein level for all four wild-type lines in the presence of the inhibitor (Supplementary Fig. 7d–f), supporting the hypothesis that PpARFb proteins are subject to proteasome-dependent degradation.

To determine whether protein levels were also elevated in the maize *Trf* mutants, we developed an antibody against ZmARF28. Semi-quantitative western blot analysis of whole-protein extract from vegetative seedlings revealed a statistically significant increase in ZmARF28 protein abundance in *Trf* compared with normal siblings (Fig. 4c,d), supporting the hypothesis that ZmARF28 is stabilized in *Trf*. To test whether ZmARF28 was also subject to proteasome-dependent degradation, we expressed wild-type and *Trf* versions of ZmARF28-GFP in *Nicotiana benthamiana* and maize protoplasts treated with the proteasome inhibitor MG132 (ref. ^51^). Western blots revealed accumulation of ZmARF28 protein in the presence of MG132, which was more marked in the wild-type ZmARF28-GFP samples (Fig. 4e and Supplementary Fig. 2i). This suggests that both the *Trf* and moss mutants disrupt a deeply functionally conserved class-B ARF region required for proteasome-dependent degradation.

To further test the effect of the *Trf* mutation on protein stability in flowering plants, we expressed the Arabidopsis orthologue of *ZmARF28, AtARF2*, and a *Trf* variant of *AtARF2* (arf2^T298N^) in Arabidopsis mesophyll protoplasts using a ratiometric reporter system. Protoplasts expressing *AtARF2* or *arf2*^T298N^ were treated with the transcriptional inhibitor cycloheximide (CHX). Similar to AtARF1 (ref. ^52^), AtARF2 shows a half-life of ~4 h. Conversely, arf2^T298N^ only lost 23% of its initial protein level after 4 h of CHX treatment, suggesting that its half-life is longer than that of wild-type AtARF2 (Fig. 4f). These results show that the *Trf* mutation in AtARF2 results in increased protein accumulation not due to increased rates of translation but more likely due to increases in protein stability, further supporting functional conservation of this region.

To test whether the mutations disrupt ubiquitylation of the domain, we immunoprecipitated wild-type and mutant versions of the PpARFb2 DBD from bortezomib-treated *proPpARFb2:DBD*^*WT/mut*^*-YFP* moss lines and probed with an anti-ubiquitin antibody. Polyubiquitylated proteins were detected for the wild-type DBD but not for the E266K/R269Q version (Fig. 4g). Combined with protein stability findings, these data indicate that the dominant mutations in moss and maize uncovered a region required for class-B ARF degradation by the 26S proteasome.

## Discussion

The precise complement and abundance of auxin-signalling components in each cell determines auxin sensitivity and how the auxin signal is decoded to produce distinct transcriptional responses. Data across land plants show that the relative levels of class-A and -B ARFs have a key role in regulating auxin response^4,13^. We leveraged a comparative analysis of species that diverged ~500 Ma and identified an ancient regulatory mechanism that controls class-B ARF accumulation. By combining our findings with an emerging understanding of differential class-A and -B binding preferences^24,32,33^, we can start to build a model of how class-A and -B ARF function may be regulated.

Our comparative mutational analysis revealed that the level of class-B ARFs is essential for dictating cell fate decisions in land plants. Elevated class-B ARFs, as in the dominant *Pparfb* mutations, block or delay differentiation towards a leafy gametophore fate. Similarly, in maize, class-B ARF ZmARF28 accumulation probably influences meristem cell fate, leaf medio-lateral patterning and sex determination. Defects in these auxin-determined decisions lead to changes in phyllotaxy, node elongation and inflorescence architecture, while fate decisions such as the transition to flowering remain unaffected. Comparisons of the *Trf* phenotype with other known maize ARF mutants (reviewed in ref. ^43^) highlight potential diversification of ARF function in organ-specific developmental programmes in different plant lineages.

It is remarkable that, despite a lack of sequence-level conservation, the same short region within the DBD of class-B ARFs has an essential role in determining protein level across land plants. Assuming that this region interacts with a second protein, perhaps a ubiquitin protein ligase, it is possible that the degradation-regulating region and interacting protein have co-evolved during the ~500 Myr that separate maize and *P. patens*, explaining differences in sequence and response, and the inability of the maize regulatory region to be recognized in moss. This further emphasizes that, similar to class-B *ARFs* regulation by the *TAS3* *trans*-acting small interfering RNA (tasiRNA), some aspects of ARF regulation are ancient, although the outcomes can be different: in flowering plants, *TAS3* regulation of *ARF3* and *ARF4* is required for adaxial–abaxial patterning, while in *P. patens* the pathway regulates protonemal development^46,53,54^. These differences further highlight lineage-specific changes to auxin-regulated cell fate decisions.

Our work underscores the importance of protein-level regulation of ARF levels in tuning auxin response. Previous work found that proteasome regulation of class-B AtARF1 and AtARF2 stability in Arabidopsis, is dependent on AtHOOKLESS1 activity^55^. More recently, the F-box protein AtAFF1 was shown to regulate the levels and behaviour of the class-A ARFs, AtARF7 and AtARF19 (ref. ^56^). Proteasomal degradation of MpARF1 and MpARF2 in *Marchantia polymorpha* has also recently been reported^57,58^. In addition, AtARF2 has recently been identified by proximity labelling with AtCUL1, part of the E3 Ligase SKP1–Cullin–F-box complex^59^, further supporting our findings here. Additional instances of proteasome regulation of ARFs, potentially conserved across evolution, as in the case of the *Trf* and *nar* mutants, may exist. It will be interesting to see whether similar non-sequence conserved regulatory mechanisms are common in the ARF lineages.

The TIR1/AFB–Aux/IAA pathway is subject to complex regulation consistent with its essential role in diverse processes. These parallel regulatory pathways enhance auxin response robustness, and modulation probably facilitated the expansion and fine-tuning of auxin-mediated processes across land plant lineages. Comparative analyses between species, such as the work presented here, is key to the discovery of the auxin puzzle pieces and how they fit together.

## Methods

### Maize plants

The *Truffula* seed was provided by Gerry Neuffer, originally called Ts-2620 and found in an EMS-mutagenized Mo17 population. The phenotype of *Trf* was analysed in backcrosses into W22 and Mo17 advanced to generation 10. Field-grown plants were grown at UC Berkeley Gill Tract, UC Davis and UMass Amherst during summer field seasons. All plants used for molecular analysis were backcrossed into W22 10 times. Seedlings grown for RNA and DNA extractions were grown under greenhouse conditions in 30 × 45 × 2.5 cm trays with peat-free soils plus osmocote. Seedlings grown for auxin treatment tests and western blots were grown in temperature-controlled greenhouses with supplemental light in 30 × 45 × 2.5 cm trays with peat-reduced osmocote compost with added fertilizer under 16 h 25 °C/8 h 21 °C day/night cycle.

### DNA extraction

Leaf discs were collected from 80 *Trf* and *N* siblings in the W22 backcross population (at least 10 introgressions). High-quality gDNA was extracted using urea as described previously^60^. In brief, genomic DNA was extracted using a urea-based extraction method which is as follows. Tissue was ground in urea extraction buffer (4 M urea, 4 M NaCl, 1 M Tris pH 8, 0.5 M EDTA, 34 mM *n*-lauroyl sarcosine). Then an equal volume of phenol:chloroform:isoamyl alcohol (25:24:1), was added and vortexed to mix. After centrifugation, the supernatant was decanted and mixed with an equal volume of chloroform. After another centrifugation, the supernatant was decanted and mixed with 1/10th volume of 4.4 M NH_4_OAc pH 5.2 and 0.7 volume of isopropanol. Strands of DNA were then collected and washed with 70% ethanol in a separate tube. The dried pellet was resuspended in 100 µl of Tris-EDTA buffer (TE). DNA integrity was checked using gel electrophoresis and quantified using Qubit before sending for library preparation and 150-bp paired-end sequencing by Novogene. Sequencing was for 10× coverage of the maize genome. DNA for SSR mapping and genotyping was extracted from 1.5 cm of leaf tissue using the protocol described previously^61^.

### Whole-genome sequencing bulk-segregant analysis

Sequence data were downloaded via ftp from Novogene servers and passed through the following analysis pipeline: FASTPQ^62^ > Bowtie2 (ref. ^63^) alignment to the B73_Reference_Nam5.0 genome > samtools mpileup variant calling^64^ > SNPeff^42^ and variant filtering in R, restricting to EMS-type SNPs. Jupyter notebooks containing the code for WGS-BSA analysis is available on GitHub at https://github.com/ThePlantShapeLab.

### SSR mapping and genotyping maize plants

SSR markers were identified using the Maize GDB database and used to genotype *Trf* phenotype plants from successive generations (see Fig. 2 for position, and Supplementary Table 1 for primers). Custom SSR markers were identified and designed to refine the region further (Supplementary Table 1). Genotyping PCRs used AD_4 and AD_5. All PCRs were carried out in 20 µl reactions using Go*Taq* Green Master Mix (Promega), following manufacturer instructions. PCR reactions were carried out on a Bio-Rad T1000 thermocycler using the following conditions: 98 °C for 5 min, then 35 cycles of 98 °C for 2 min, 58 °C for 30 s, 72 °C for 30 s, then 72 °C for 10 min. The PCR product (10 µl) was analysed using thin metaphor agarose (3.5% in 1× TBE, 1:10,000 SYBR safe, 90 V, 1 h) electrophoresis and imaged on a BioRad GelDoc GO imaging system.

### Auxin treatment of maize

A segregating population of 5-week-old *Trf* siblings was genotyped using primers AD_4 and AD_5. Plants were cut at the root–shoot node and submerged in either 100 µm indole-3-acetic acid (Sigma, I5148) in 1% ethanol, or 1% ethanol only, ensuring that at least 10 cm of the shoot base was completely submerged. Shoot apices were harvested (~1 cm encompassing the base of the meristem and young leaf tissue, outer leaf removed), dried briefly and flash frozen in liquid nitrogen at 0 min and 30 min. Two replicate pools for each treatment and genotype group were collected, containing 3 individuals in each pool.

### Maize RNA extraction

For the *Trf* versus normal (N) sibling RNA-seq experiment, a segregating population of 3-week-old maize seedlings were first genotyped for *Trf* (see Supplementary Table 1 for primers). The outer 2 leaves were removed and 0.5 cm of the shoot apex was dissected and flash frozen in liquid nitrogen. Three replicate pools were created for *Trf* and N siblings, with 10 individuals per pool. For the auxin treatment of *Trf* versus N siblings, tissues were treated as described above. RNA for all experiments was extracted from each pool using TRIzol (Invitrogen) as described in the manual. RNA was DNAse treated to remove gDNA contamination using Invitrogen DNaseI (18068015) digestion. RNA quality was checked using gel electrophoresis (1× Agarose in 1× TBE) and quantified using Qubit before sending for library preparation and 150-bp paired-end sequencing by Novogene, or generating cDNA libraries using the iScript gDNA clear cDNA synthesis kit (Bio-Rad).

### RNA-seq analysis

Sequence data were downloaded via ftp from Novogene servers and passed through the following analysis pipeline: FASTPQ^62^ > HiSAT2 (ref. ^65^) alignment to the *Zea mays* Reference NAM5.0 genome > FeatureCounts^66^ > DeSeq2 (ref. ^67^) analysis of differential gene expression using genes with more than 5 counts in 3 or more samples. All analysis was carried out locally (3.6 GHz 8-Core Intel Core i9 processor, 32 GB RAM) using R packages combined with Jupyter notebooks. Jupyter notebooks containing the code for RNA-seq analysis are available on GitHub at https://github.com/ThePlantShapeLab. GO term analysis was carried out using the GAMER Maize annotations^68^ and the GOSeq R package^69^; significantly enriched GO terms had a Benjamini and Hochberg cut-off *P*_adj_ value of <0.05. Comparisons of significantly differentially expressed genes (*P*_adj_ < 0.05, log_2_FC < −0.5 or >0.5) with potential class-B ARF regulatory targets were carried out using the DAP-seq data published in ref. ^24^ aligned to the maize B73 NAM5.0 genome (provided by Andrea Gallavotti and Mary Galli, Rutgers University). DAP-seq peak data for three class-B ARFs in the same subclade as ZmARF28: ZmARF13 Zm00001eb170540, ZmARF10 Zm00001eb142540 and ZmARF25 Zm00001eb363810 were used (ZmARF28 does not have any published DAP-seq data). DAP-seq target genes were filtered and considered ‘high-confidence’ targets if the DAP-seq peak was within 1 kb of the transcriptional start site, generating a list of 4,654 class-B target genes, of which 61% are shared.

### cDNA synthesis and qPCR analysis

cDNA was synthesized from 750 ng RNA using the iScript gDNA clear cDNA synthesis kit (BioRad) according to the manufacturer’s manual. cDNA was then diluted 1:10 for subsequent analysis. Primers for qPCR were designed spanning intron/exon boundaries for auxin-responsive gene *ZmSAUR27* (Zm00001eb104110, primers AER639:CTGGAGGGTGGAAAGGGAAG, AER640:AGCATCAAAAGGCTCCATGGA) and the GAPDH housekeeping gene (Zm00001eb173410, primers AER647:CATCATTCCTAGCAGCACCG, AER648:TACACAAGCAGCAACCATCC) using Benchling and checked for specificity using the Maize GDB BLAST tool (https://www.maizegdb.org/). Primers were then tested using standard RT–PCR conditions (1 µl 1:10 cDNA, 2 µl of each 5 µM primer, 10 µl Go*Taq* Green Master Mix, 5 µl nuclease-free H_2_O, run on BioRad ThermoCycler T2100 30 cycles of 95 °C/58 °C/72 °C) and evaluated using gel electrophoresis (1% agarose in TBE, 90 V, 40 min). All primers generated single, specific bands at the expected size (~500 bp). To confirm that identity bands were excised, the DNA was extracted using Qiagen Gel Extraction kit and sent for Sanger sequencing. qPCR was performed with the optimized primers using PowerUp SYBR Green Master Mix (Applied Biosystems) according to manufacturer instructions, and the Applied Biosystems StepOnePlus Real-Time PCR System. Optimum conditions were determined as follows: first, primer specificity was tested by a melting curve analysis, yielding a single peak per primer pair. Next, serial dilutions of each sample were used to determine the optimum cDNA concentration, which was found to be the same 1:10 dilution as above, with a 20 µl final volume for each reaction. Reactions were carried out as follows: a hold step (2 min, 50 °C) and initial denaturation (2 min, 95 °C); 40 cycles of 95 °C (15 s) followed by 60 °C (1 min); ending with a melt curve (60 to 95 °C). The ${{2}{^{-{\Delta\Delta}{C}_{T}}}}$ method was used to calculate relative quantification^70^, with values normalized to the average of the mock treatment for the WT pools.

### Antibody production

Antibody was developed from the ZmARF28 full-length coding sequence cloned into pDEST17 bacterial destination vector carrying the 6His tag for antigen production. Recombinant protein expression was carried out in *E. coli* Rosetta strain (BL21). After purification, a total of 200 µg of protein was sent to Cocalico Biologicals where two guinea pig immunizations were performed. To improve specificity of this antibody, purification from antiserum was carried out using a synthetic 20-amino-acid peptide from position 505 to 524 which is a highly specific region to ZmARF28 (amino acids 505–524: RPFPNKISGTRSSTWVTADA) using magnetic beads from Invitrogen. A cysteine (C) residue was added at the N-terminal end of the peptide to enable covalent binding to the beads used for antibody purification. To validate specificity of the purified antibody, a western blot was carried out following the described protocol in the next section, using 20 mg of soluble total protein from vegetative B73 maize shoot apical meristem.

### ZmARF28 protein extraction from maize and western blots

Maize seedling (3-week-old) shoot apices were harvested and flash frozen in liquid nitrogen before grinding to a fine powder. Powdered tissue (100 mg) was then thawed in 1× SDS + dithiothreitol loading buffer and vortexed. Samples were heated at 95 °C for 5 min before loading on a 10% acrylamide gel for electrophoresis. Semi-dry transfer to a methanol-activated PVDF membrane in 1× Tris-glycine/20% methanol was carried out. Membranes were air dried, reactivated in methanol, washed in TBS/0.05% Tween for 5 min and then blocked for 1 h with 6% skimmed milk in TBS/0.05% Tween at room temperature. Membranes were incubated overnight in the primary anti-ARF28 antibody diluted 1:250 in 1% milk/TBS/0.05% Tween. The following day, membranes were washed (3× 5 min in TBS/0.05% Tween) and incubated with 1:5,000 anti-guinea pig-HRP secondary antibody (Thermo Fisher, A18769) at room temperature with rocking for 2 h. After washing (4× 5 min in TBS/0.05% Tween), membranes were incubated with Clarity Western ECL substrate (Bio-rad, 1705060) and exposed to X-ray film. Film was exposed to the membrane for 10 min before developing. This experiment was repeated four times with similar results.

### Expression of *ZmARF28-GFP* in *N. benthamiana* and western blots

The CDS of ZmARF28 and ZmARF28-*Trf* was cloned into pEARLYGATE103 using gateway cloning. Sequence was confirmed using plasmid nanopore sequencing (Source Biosciences). The ZmARF28-pEARLYGATE103 constructs were transformed into *Agrobacterium* strain AGL0, and successful transformants were confirmed using colony PCR. Glycerol stocks of colonies were created and used for all subsequent experiments. Overnight cultures of *Agrobacterium* strains containing ZmARF28-pEARLYGATE103, ZmARF28*-Trf-*pEARLYGATE103 or pEARLYGATE103 empty vector were grown in Luria–Bertani (LB) media with 10 µg ml^−1^ rifampicin and 50 µg ml^−1^ kanamycin at 28 °C with 200 r.p.m. shaking. The next day, the optical density at 600 nm (OD_600_) of the cultures was measured and the cultures diluted in infiltration buffer (10 mM MES, 10 mM MgCl_2_, 0.2 mM acetosyringone) to OD_600_ = 0.3 for pEARLYGATE103 constructs. Leaves of *N. benthamiana* plants at 2 weeks post transplantation to soil were infiltrated with a pEARLYGATE103 strain and then left to grow for a further 2 days (16 h 22 °C/8 h 22 °C day/night cycle). Infiltrated leaves were then infiltrated with 5 µl 100 µM MG132 in DMSO, or DMSO only, diluted in 1 ml infiltration buffer and incubated for 4 h before harvest and snap freezing in liquid nitrogen. Leaves were ground to a very fine powder in liquid nitrogen before adding extraction buffer (50 mM NaCl, 1% Igepal CA-630, 0.5% sodium deoxycholate, 0.1% SDS, 50 mM Tris-HCl (pH 8.0), 1 mM EDTA, 50 mM NEM, 1× cOmplete EDTA-free protease inhibitor cocktail (Roche, 11836170001), 1× PhosSTOP phosphatase inhibitor (Roche, PHOSS-RO)) and continuing to grind until defrosted. Samples were transferred to Eppendorf tubes and centrifuged at 6,500 × *g* at 4 °C for 12 min. The supernatant was transferred to a new Eppendorf tube and centrifuged at 9,800 × *g* at 4 °C for 3 min. Protein was quantified using a Qubit Broad Range Protein kit and the Qubit fluorometer 4 (Invitrogen). Total protein extract was diluted to 0.5 µg ml^−1^ in extraction buffer. A volume of 10 µl of extract was mixed with 4× SDS + dithiothreitol (5× buffer stock: 0.25% bromophenol blue, 0.5 M dithiothreitol, 50% glycerol, 10% sodium dodecyl sulfate, 0.25 M Tris-HCl pH6.8) loading buffer and heated at 95 °C for 5 min. Sample (20 µl) was loaded onto a 12% SDS–PAGE gel and ran in 1× Tris-glycine running buffer with 0.1% SDS at 70 V for 30 min, then 100 V for 90 min at room temperature. Proteins were transferred overnight at 4 °C using the Bio-Rad wet-transfer mini-blot system to a PVDF membrane. Membranes were washed in dH_2_O and dried before reactivating in methanol for 1 min and washing in dH_2_O. Membranes were then stained for 5 min in Ponceau stain, and blots were imaged after 3 rinses in dH_2_O. Ponceau stain was washed off the blot using 1× PBS-T (1× PBS, 0.05% Tween-20). Membranes were blocked at room temperature for 2 h in 5% milk/PBS-T with shaking, then incubated with 1:3,000 mouse anti-GFP (Roche, 11814460001) for 90 min, washed 3 times in 1× PBS-T, then incubated with 1:3,000 anti-mouse-HRP (Cell Signaling Technology, 7076) for 1 h at room temperature. Blots were washed three times in 1× PBS-T before incubating with SuperSignal West Pico PLUS Chemiluminescent Substrate (Thermo Scientific, 34577) for 1 min according to manufacturer instructions and imaged on an Azure 300 Chemiluminescent imager. Experiments were repeated 3 times.

### Expression of ZmARF28-GFP in maize protoplasts

Protoplasts were extracted as follows. A 3–4-week-old seedling was harvested and the outer unexpanded leaves removed before being cut into 2-cm sections and surface sterilized in 0.5% bleach/1% Tween-20 for 3 min. Tissue was then rinsed in sterile water four times and patted dry using kimwipes. Tissue was finely sliced (<1 mm) in a sterile glass Petri dish and 10 ml of sterile enzyme solution added (10 mM KCl, 8 mM MES, 1 mM CaCl_2_, 0.5 M mannitol, 0.6% cellulase RS, 0.1% macroenzyme R-10, 0.1% bovine serum albumin, 0.1% polyvinylpyrrolidone K30) before incubating on an orbital shaker (20 mm orbit, 40 r.p.m.) for 2 h at room temperature in the dark. A volume of 10 ml sterile W5 solution (154 mM NaCl, 125 mM CaCl_2_, 5 mM KCl, 2 mM MES) was then added and incubated for 1 h at room temperature on an orbital shaker (20 mm orbit, 80 r.p.m.) in the dark. This was then filtered through a 70-µm cell strainer before centrifugation at 100 × *g* for 5 min. Protoplasts were resuspended in 1 ml suspension solution (0.4 M mannitol, 20 mM CalCl_2_, 5 mM MES) and viability checked using fluorescein diacetate (FDA) staining and confocal microscopy. Protoplasts (200 µl) at a concentration of 400,000 per ml were then incubated with 20 µg of plasmid DNA (full-length ZmARF28 or ZmARF28-*Trf* in pEARLYGATE103) for 5 min at room temperature. A volume of 220 µl PEG solution (40% PEG 4000, 0.1 M CalCl_2_, 0,4 M mannitol pH 5.7, 50 mM MES) was added and mixed by inversion before incubation at 28 °C for 15 min. PEG was diluted with 800 µl of W5 solution, protoplasts were pelleted (100 × *g*, 3 min), washed with 500 µl W5 solution and then resuspended in 300 µl incubation buffer (0.5 M mannitol, 4 mM KCl, 4 mM MES, 1% BSA, 50 µg ml^−1^ kanamycin) and incubated overnight at room temperature in the dark on an orbital shaker (20 mm orbit, 20 r.p.m.). Protoplasts were checked for GFP expression using confocal microscopy before proceeding with MG132 treatment (20 µM MG132 or DMSO mock treatment) and incubated for a further 3 h. Once complete, protoplasts were pelleted and washed with W5 solution before proceeding with protein extraction and western blots as described above for the *N. benthamiana* protein expression. Experiments were repeated 3 times.

### Image analysis and figure assembly

All images were analysed in FIJI^71^. Figures were assembled using Adobe Photoshop.

### Statistical analyses

All data were tested for normality using the Shapiro–Wilk test. On the basis of this analysis, data with a normal distribution were analysed using a Student’s *t*-test or analysis of variance (ANOVA) where appropriate. Data that were not normally distributed were analysed using the non-parametric Kruskal–Wallis test, followed by pairwise Wilcox tests. A Jupyter notebook containing the code for data analysis is available on GitHub at https://github.com/ThePlantShapeLab.

### Moss growth

Moss was grown on BCD minimal medium or BCD supplemented with either 5 mM ammonium tartrate (BCDAT) or 5 µM NAA (BCD + NAA)^72^. BCDAT was used for routine growth. Ammonium directly or indirectly dampens auxin responses, which enhances the phenotypes of mutants with reduced auxin response. For growth assays, ~1-mm^2^ pieces of tissue from chloronema-rich cultures of each genotype were spotted in 4–6 different positions on the plates containing all three media, with each genotype represented by at least two independent lines. The *nar* mutants were isolated in the *DR5:DsRed/NLS-4* background in the Gransden ecotype^13^, and all subsequent analyses were carried out in either the Reute ecotype or a strain with the *DR5:DsRed* transgene introgressed from the semi-fertile Gransden strain into Reute through three crosses. During the course of this work, it was discovered that the *DR5:DsRed* expression is often silenced upon retransformation, possibly due to the locus containing approximately 60 repeats of the transgene^45^.

### Moss mutant screen

The screen for *nar* mutants and mutant genome sequencing was described previously^39^. Briefly, 30 plates of week-old lawns of *DR5:DsRed/NLS-4* on cellophane-overlain BCDAT media were irradiated with 150 mJ cm^−2^ of UV light in a Stratalinker UV Crosslinker (Stratagene). After 24 h in darkness, the cellophanes were moved to new BCD + NAA plates, and the mutants were identified after 2–4 weeks. Confirmed mutants were screened for mutations in *Aux*/*IAA* and *DIAGEOTROPICA* genes by sequencing PCR products, and genomes of 12 of the strongest remaining mutants were sequenced. The full set of variants in the mutant genomes were filtered for those causing protein-altering changes in the same gene in two or more of the mutants. While both *narC29* and *narC18* had the E263K substitutions in *ARFb4*, *narC18* also had a G608S substitution in class D ARF, *ARFd1*, which probably accounts for the stronger phenotype. For simplicity, only *narC29* is shown.

### CRISPR/Cas9

Plasmids for expressing one or two single guide RNAs (sgRNAs) and Cas9 were assembled either as described in ref. ^73^ or after modification to bypass the Gateway cloning step: the empty pENTR-L1L2-U6:BsaI insert was recombined into the pMH-Cas9-Gateway vector with LR Clonase II (Invitrogen), then the resulting plasmid backbone was replaced with that of pUK21 which lacks BsaI restriction sites and thus allows GoldenGate assembly of protospacers between the BsaI sites after the PpU6 promoter. A fragment of GFP was inserted between the BsaI sites to help distinguish recombinant clones from the parental vector. The *hph* selection gene was swapped for *nptII*, *aacC1* or *ble* to allow selection with kanamycin/G418, gentamicin/G418 and Zeocin, respectively, in addition to Hyg. Protospacers were selected on the basis of predicted specificity and efficiency using the CRISPOR website (http://crispor.gi.ucsc.edu/)^74^. sgRNAs starting with G were preferred. Plasmids for making deletions contained dual *PpU6:sgRNA* genes targeting shortly after the start codons and before the stop codons. Oligo-mediated gene editing was achieved by designing double-stranded oligos matching the target but with the intended missense mutation(s) and silent mutations to create a restriction site and multiple mismatches in the PAM-proximal half of the sgRNA target. The oligos extended 21 bases before and after the mismatched bases.

### Moss transformations

Protoplasts were prepared from week-old tissue and transformed as previously described^72^, except that the post-heat-shock dilution was done by slowly adding 10 ml 8.5% mannitol all at once rather than 6.5 ml in 10 steps. Transformations included 15–25 µg of each plasmid and 250 pmol of annealed oligos. Deletion lines were identified by dramatically truncated PCR products and Sanger sequencing. Genome-edited lines were identified by PCR across the edited site, followed by digestion with the restriction enzyme whose site was added to the template oligos. PCR products with the expected digestion pattern were sequenced. In several instances, lines exhibiting the same mutant phenotype but lacking the restriction site change or lacking the phenotype but having the restriction site change were found to have incorporated either the missense mutation or the restriction site, respectively, without the other. In the first editing experiment to re-create the E266K and R269Q mutations in *PpARFb2*, it was discovered that the *proDR5:DsRed* transgene’s gentamicin selection cassette also confers resistance to the G418 selection agent and this precluded selection of the CRISPR plasmid. We instead screened the transformants for resistance to NAA. Some of the NAA-resistant lines had the intended mutation but many had small in-frame insertions and deletions in the region.

YFP-tagging of endogenous loci was achieved by co-transforming a CRISPR plasmid targeting near the stop codon and a plasmid that included: 600–1,500 bp homology arm of the coding region preceding the stop codon fused in-frame to a linker (SRGGGGA) and *YFP* (encoding a bright, monomeric yellow fluorescent protein), followed by the *Pisum rbcS* terminator, a *pro35S:Hyg:ter35S* selection cassette flanked by *loxP* sites and a downstream homology arm consisting of the 3′ UTR and downstream sequences. Stable lines were identified by selecting for the CRISPR plasmid and the knock-in construct for 7 days, followed by 10–14 days of no selection and 7 days of Hyg selection. Clean insertion lines were identified by (1) PCR amplification with primers matching before the upstream homology arm and downstream from the downstream homology arm with primers to *YFP* and *ter35S*, and (2) lack of PCR amplification both across the insertion site and of the Cas9 gene. In the case of the *pparfb1*^*E266K*^ and *pparfb2*^*E266K*^, the strength of the phenotypes increased upon integrating the *YFP*/*Hyg* sequences. Removal of the *pro35S:Hyg:ter35S* selection cassette by *CRE*/*lox*-mediated excision reverted the phenotype to that before insertion, suggesting that *pro35S* enhancers may act at a distance on the nearby promoters in moss as in Arabidopsis^75^. All YFP lines shown had their selection cassettes excised.

The *proARFb2:DBD-YFP* constructs with and without the E266K + R269Q substitutions and the overexpression constructs were constructed by GoldenGate assembly. The *DBD-YFP* assembly included plasmids containing mYPet and 3 kb of sequence upstream from *ARFb2*’s start codon, PCR products containing the DBD sequences before and after the loop sequence, double-stranded oligos with the wild-type or mutant loop sequences and the pMP2133 destination vector. The pMP2133 vector contained *PIG1*-locus-homology arms for targeting, *AtHsp18.2* terminator sequences and a *pro35S:nptII:ter35S* selection cassette. The overexpression construct assembly included the pMP2280 destination vector, plasmids containing the maize *Ubiquitin* promoter, the *PpARFb2* or *ZmARF28* cDNAs (without stop codons), and a double-stranded oligo with a C-terminal 1× Myc epitope and a stop codon. The pMP2280 vector contained *Pp108* homology arms flanking the cloning site, a *nos* terminator and a *pro35S:Hyg:ter35S* selection cassette. The constructs were co-transformed into Reute WT along with a CRISPR plasmid targeting the corresponding native neutral targeting locus. Transformants were selected for 7 days with hygromycin and G418, followed by 10 days off selection. Stable lines were identified by PCR after screening lines resistant to G418 and sensitive to hygromycin (DBD lines), or resistant to hygromycin (overexpression lines).

### Moss RT–PCR

Week-old *pparfb2*^*E266K*^, *ppiaa2*^*G325S*^ and the corresponding wild-type (*DR5:DsRed*/3×Re) tissues were homogenized, plated on BCDAT plates overlain with cellophanes and grown for 6 days. At 18 h before treatment, the cultures on cellophanes were moved to BCD plates to alleviate the antagonism on auxin response by ammonium. Approximately 100 mg of tissue were scraped from the cellophane and transferred in triplicate to wells of 6-well plates with BCD-agar media containing either 10 µM IAA or 0.01% ethanol with 1 ml liquid BCD media, with the same concentrations of IAA and ethanol. After 1 h, tissue was patted dry between paper towels, and RNA was extracted using the Quick-RNA Plant Miniprep kit (Zymo Research) and a Mini-Beadbeater-16 (BioSpec Products) according to manufacturer instructions. Total RNA (1.3 µg) was reverse transcribed using the Maxima H Minus cDNA Synthesis Master Mix (Thermo Fisher), diluted 8-fold with 0.1× TE, and 3 µl used per 20 µl SYBR-qPCR reaction using the CFX Connect-96 Real-Time PCR Detection System (Bio-Rad). Data represent two technical replicates for each of the three biological replicates for each genotype and treatment.

### Yeast two-hybrid assay

Yeast interaction assays were carried out using the Matchmaker LexA Two-Hybrid System (Clontech). Interactions were indicated by β-galactosidase detection on SD/Gal/Raf/X-Gal plates. For testing BRD function, a C-terminally Myc-tagged full-length TPL2/Pp6c9_10690 cDNA was inserted into pGilda (LexA DBD), and full-length ARFb2 cDNA with and without the L614S and F615S substitutions were inserted into pB42AD. No dimerization was detected between DBD/DD domains using pGilda+ARFb2[aa1–375] and pB42AD + ARFb2[full-length].

### Moss imaging

For growth assays, 3-week-old moss were photographed with a Nikon SMZ1500 and a DS-Ri1 camera. DsRed fluorescence was captured using a TRITC filter set. Confocal microscopy was performed using a Zeiss LSM880. Tissue was mounted in water or liquid BCD medium, and images were captured for differential interference contrast (DIC), YFP fluorescence and chlorophyll autofluorescence. FIJI was used for processing images and for measuring DsRed and YFP signals^71^. DsRed signal was measured from oval selections of whole plants and regions of background and calculated as IntDen[sample]−(IntDen[background]×Area[sample]/Area[Background]). For YFP, nuclei were identified in the YFP or DIC channels, circled as freehand selections and their integrated density measured. Note that not all nuclei in wild type, unlike the mutants, have detectable YFP signal and thus could not be measured, hence their quantification is probably an overestimate and the difference between wild type and the mutants is an underestimate. The *proARFb2:DBD-YFP* lines were mounted in water and imaged with a Keyence BZ-X810 microscope with an EYFP filter cube and a ×4 objective to capture brightfield and YFP (2 s exposure time) images.

### Detection of ubiquitylated protein

Tissue from untransformed Reute WT (Re), *proARFb2:DBD-YFP* (T215-5, DBDY) and *proARFb2:dbd-YFP* (E266KR269Q, T216-2, dbdY) strains were homogenized and split into 10 plates of BCDAT medium overlain with cellophane sheets and grown for 9 days. Moss tissues (200–250 mg) were added to wells of 6-well plates containing solid BDCAT + 100 µM bortezomib (LC Laboratories, B-1408) plus 1 ml liquid BCDAT + 200 µM bortezomib. After 18 h, tissues from 3 wells (Re and DBDY) or 1 well (dbdY) were patted dry between paper towels and frozen in liquid nitrogen in triplicate. Frozen tissue was ground to a powder while frozen, then mixed with 1,350 µl (Re, DBDY) or 450 µl (dbdY) chilled lysis buffer (50 mM Tris-HCl pH 7.5, 150 mM NaCl, 1% Ipegal CA-630, 50 mM *N*-ethylmaleimide, 1.75× plant protease inhibitor cocktail (Millipore, P9599), 50 mM NaF, 10 mM β-glycerophosphate) and transferred to microfuge tubes in ice. After pelleting debris at 17,000 *g* for 10 min, ‘input’ samples were removed and 25 µl of thrice-washed GFP-Trap magnetic beads (Bulldog Bio, GMA020) were added to each tube and rotated for 2 h at 4 °C. The beads were gathered using a magnetic stand, and the liquid was removed and replaced with 1 ml wash buffer (50 mM Tris-HCl pH 7.5, 150 mM NaCl, 0.05% Ipegal CA-630). Three washes in total were performed, with the beads being transferred to clean tubes in the second wash. The third wash was completely removed and replaced with 70 µl of 2× SDS sample buffer, mixed and heated to 95 °C for 10 min before removing immunoprecipitated (IP) samples from the beads. Samples were separated using 7.5% SDS–PAGE gels transferred to nitrocellulose membranes using the Bio-Rad wet-transfer mini-blot system. Membranes were rinsed with TBST and blocked for 2 h in 5% milk, then incubated overnight at 4 °C with the primary antibodies in 5% BSA. GFP was detected using the rabbit polyclonal antibody (Invitrogen, A-11122, 1:2,000 dilution), and ubiquitin was detected with the P4D1 mouse monoclonal antibody (Cell Signaling Technology, 3936T, 1:1,000). Blots were washed 3× in TBST and incubated for 2 h in 5% milk with the goat-anti-rabbit (Millipore, A6154) or goat-anti-mouse (Bio-Rad, 1706516) peroxidase-conjugated secondary antibodies. After 3 washes, the blots were incubated with SuperSignal West Pico PLUS Chemiluminescent Substrate for 5 min and imaged using an ImageQuant LAS 4000 mini-imager. For the IP samples, equivalent amounts of Re and DBDY elution samples were loaded along with a 1:5 dilution of the dbdY samples to give equivalent amounts of YFP fusion protein. This ratio was estimated from preliminary blot of the IP samples.

### Cloning ARF2 and arf2T98N constructs

DNA encoding 4 GS repeats, a porcine teschovirus-1 2A (P2A) cleavage peptide^76^ and mScarlet3 were synthesized using TWIST biosciences fragment synthesis. The P2A-mScarlet3 insert was amplified by PCR using Q5 2× master mix (NEB). The protoplast expression vector pLCS107 was linearized by PCR using Q5 2× master mix (NEB). The insert was cloned into linearized pLCS107 using NEBuilder Hifi cloning reactions to generate pUBQ-mNEON-P2A-mScarlet. The arf2T298N variant was introduced into the ARF2 CDS in pENTR by PCR amplification and infusion cloning (Takara BioSciences). Both wild type and T298N ARF2 were amplified from a pENTR backbone by PCR and cloned in-frame with mNEON and the GS linker using NEBuilder Hifi cloning. The pUBQ-mNEON-2A-mScarlet backbone was linearized by PCR.

### Arabidopsis protoplast expression and quantification

Arabidopsis mesophyll protoplasts were isolated from 14-day-old Col-0 leaves. Cells (100,000) were transformed with 20–30 μg of plasmid DNA carrying mNEON-ARF2-P2A-mScarlet3 or mNEON-arf2T298N-P2A-mScarlet3 constructs using the tape-sandwich method and incubated for 16 h in the dark^77^. Transformed cell populations were scored using the Beckman Coulter Cytoflex S flow cytometer and CytExpert software. A back gating strategy was taken to identify the population of intact protoplasts. Cells expressing mScarlet3 reporters were first identified by comparing transformed mNEON-ARF2-P2A-mScarlet3 cells to untransformed cells using the Y610 channel (561 nm excitation, 610 ± 20 nm emission, 1,000 gain) and then back gated on FSCvSSC. Cells were treated with 100 µM cycloheximide diluted in protoplast media for the indicated times. A minimum of 162 mScarlet3 positive cells from 6 independent transformations were used to collect mScarlet3 reporter levels. The mNEON levels were collected using the B525 channel (488 nm excitation, 525 ± 40 nm emission, 69 gain). FCS files for mScarlet3 positive cell populations were generated by Cytoflex and analysed using FlowKit^78^, NumPy^79^ and Pandas^80^ (https://zenodo.org/record/7979740) packages in Python with custom scripts. Ratios were determined by taking the mNEON signal divided by the mScarlet3 signal on a per cell basis. The mean ratio of each replicate at timepoint 0 was generated and all cells from that replicate were normalized to this value. A minimum of 2,166 cells across all 6 replicates were collected. Graphs were generated using seaborn^81^ and Matplotlib^82^ in Python.

### ARF DNA-binding domain structural predictions

Protein structure predictions of ARF DNA-binding domains as defined in ref. ^21^ from wild-type and mutant ZmARF28, PpARFb2 and PpARFb4 were generated using CollabFold v.1.5.2 (https://colab.research.google.com/github/sokrypton/ColabFold/blob/main/AlphaFold2.ipynb)^83^ accessed on 8 August 2023 using default conditions. The top model from each prediction was visualized with PyMol 2.5 (http://www.pymol.org). AlphaFold pTM values are shown in Supplementary Table 10.

### Phylogenetic tree construction

Protein sequences for class-B ARFs from 13 species were identified by reciprocal BLAST searches and aligned using T-COFFEE (v.11.00.8cbe486)^84^. Non-conserved regions were trimmed using Mesquite (v.3.70)^85^, and a tree was inferred using MrBayes (v.3.2.7)^86^ with the settings ‘aamodelpr=mixed, rates=invgamma, 2 runs of 4 chains for 1,000,000 generations’.

Databases used were for the following species: *Alsophila spinulosa*^87^, *Amborella trichopoda*^88^, *Arabidopsis thaliana*^89^, *Ceratodon purpureus*^90^, *Ceratopteris richardii*^91^, *Diphasiastrum complanatum*^92^, *Marchantia polymorpha*^93^, *Physcomitrium patens*^94^, *Selaginella moellendorffii*^95^, *Solanum lycopersicum*^96^, *Takakia lepidozioides*^97^, *Thuja plicata*^98^ and *Zea mays*^99^. Class-B ARF sequences were found neither in the three published genomes nor in the 1KP transcriptomes for hornwort species^100–102^.

### Reporting summary

Further information on research design is available in the Nature Portfolio Reporting Summary linked to this article.

## Supplementary information

Supplementary Information Supplementary Figs. 1–7, legends for Supplementary Tables 1–10 and unprocessed western blots for Supplementary Fig. 2I.

Reporting Summary

Supplementary Tables Supplementary Tables 1–10.

## Source data

Source Data Fig. 4 Unprocessed western blot images for Fig. 4c,e,g.

## Data Availability

All original numerical, sequence and image data are included in the manuscript or freely available via the University of Edinburgh DataShare service at 10.7488/ds/7903 (ref. ^103^); mutant and parental *P. patens* raw sequence reads are available at BioProject (PRJNA1210789); raw sequence reads for the *Zea mays* work is available for download at NCBI GEO accession numbers GSE293375, GSE293431, GSE293432. Seeds and plant material are available on request from A.E.R. (maize), M.E. (*P. patens*) and L.C.S. (*Arabidopsis*). Source data are provided with this paper.

## Code availability

All code used for the maize sequence analyses is available on GitHub at https://github.com/ThePlantShapeLab/Truffula (ref. ^104^).
