# Supplementary material for: Comparative mutant analyses reveal a novel mechanism of ARF regulation in land plants
Source: Nat Plants. 2025 Apr 11;11(4):821–35. doi: 10.1038/s41477-025-01973-3 (PMC12014491; doi:10.1038/s41477-025-01973-3)

nature plants

Supplementary information https://doi.org/10.1038/s41477-025-01973-3

Comparative mutant analyses reveal a novel mechanism of ARF regulation in land plants

In the format provided by the authors and unedited

Title: Comparative mutant analyses reveal a novel mechanism of ARF regulation in land plants

Authors: Michael J. Prigge[1], Nicholas Morffy[2], Amber de Neve[3,4], Whitnie Szutu[1], María Jazmín Abraham-Juárez[5], Trisha McAllister[6], Heather Jones[6], Kjel Johnson[3,4], Nicole Do[1], Meirav Lavy[1], Sarah Hake[3,4], Lucia Strader*[2], Mark Estelle*[1], Annis E. Richardson*[3,4,6]†

(1) Department of Cell and Developmental Biology, School of Biological Sciences, University of California San Diego, La Jolla, CA, USA

(2) Department of Biology, Duke University, Durham, NC, USA

(3) USDA Plant Gene Expression Center, 800 Buchanan Street, Albany, CA 94710.

(4) Department of Plant and Microbial Biology, University of California Berkeley, CA.

(5) Laboratorio Nacional de Genómica para la Biodiversidad (LANGEBIO), Unidad de Genómica Avanzada, Centro de Investigación y de Estudios Avanzados (CINVESTAV), Irapuato, 36821, Mexico.

(6) Institute of Molecular Plant Sciences, School of Biological Sciences, University of Edinburgh, Edinburgh, UK.

***Co-corresponding Authors:** Email **annis.richardson@ed.ac.uk**, **mestelle@ucsd.edu**, **lucia.strader@duke.edu**

† current location

Supplementary Materials

Figures S1-S7

Tables S1-S10

Unprocessed western blot images for fig.S2I

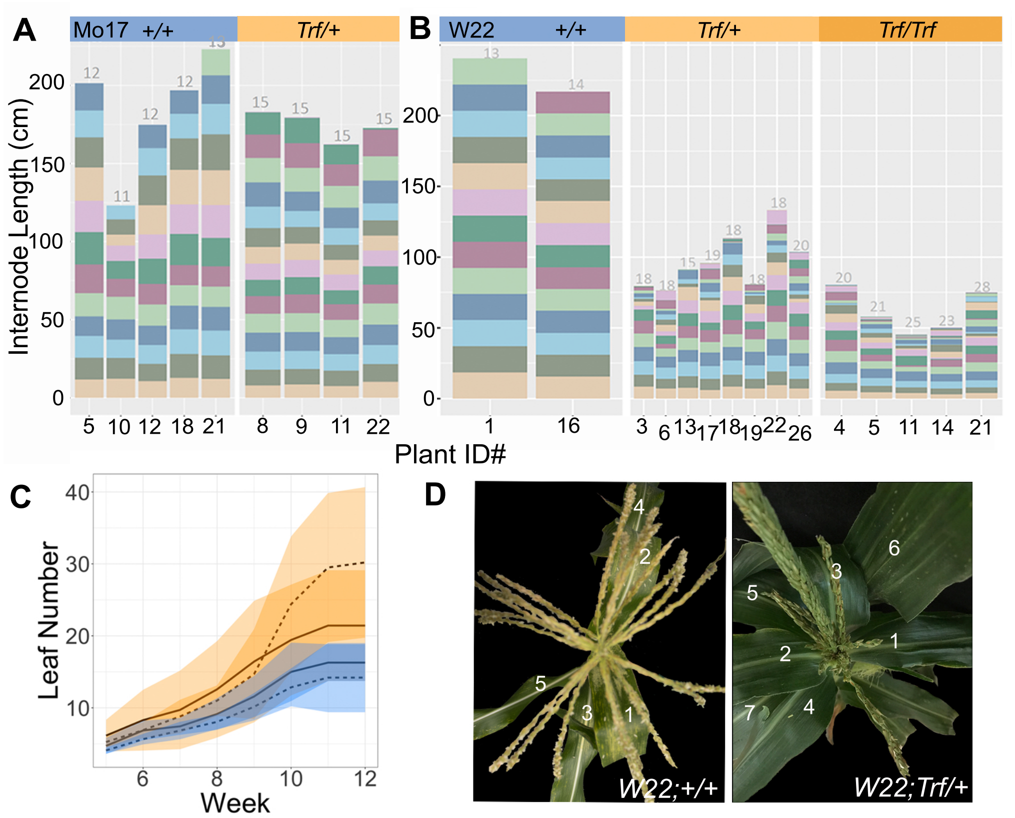

Fig. S1. *Truffula* mutants have defects in leaf initiation and internode elongation

Trf mutants (orange panels) in both Mo17 (**A**) and W22 (**B**) backgrounds have defects in internode elongation, with internode size shorter and more variable than normal siblings. Total bar height: plant height, colour bars: internode length, numbers above the columns: number of visible internodes in each mature plant. (**C**) Leaves are initiated at a faster rate in heterozygous *Trf* mutants (orange) compared to normal siblings (blue) in both Mo17 (solid line) and W22 (dashed line) backgrounds. By week 12 both *Trf* and normal siblings had mature tassels. Line: mean leaf number, colour shading: upper and lower bounds (mean ± 1.5 sd, n>3/ genotype). (**D**) *Trf* mutants have defects in leaf phyllotaxy, compared to normal siblings. Photographs of mature normal and *Trf* siblings from above, leaves are numbered in order from the tassel downwards.

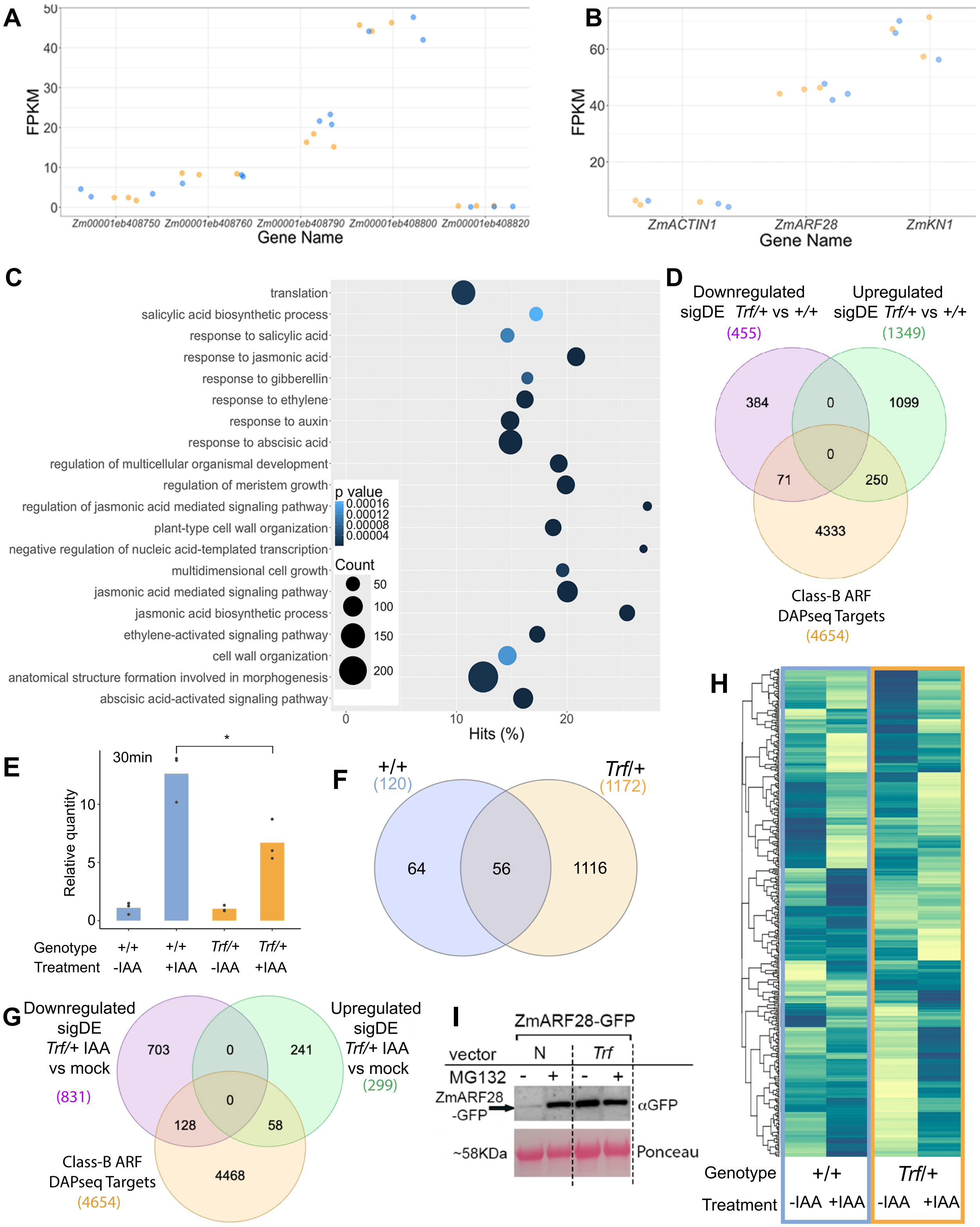
A
FPKM
Zm00001eb408750
Zm00001eb408760
Zm00001eb408790
Zm00001eb408800
Zm00001eb408820
Gene Name
B
FPKM
ZmACTIN1
ZmARF28
ZmKN1
Gene Name
C
translation
salicylic acid biosynthetic process
response to salicylic acid
response to jasmonic acid
response to gibberellin
response to ethylene
response to auxin
response to abscisic acid
regulation of multicellular organismal development
regulation of meristem growth
regulation of jasmonic acid mediated signaling pathway
plant-type cell wall organization
negative regulation of nucleic acid-templated transcription
multidimensional cell growth
jasmonic acid mediated signaling pathway
jasmonic acid biosynthetic process
ethylene-activated signaling pathway
cell wall organization
anatomical structure formation involved in morphogenesis
abscisic acid-activated signaling pathway
p value
Count
Hits (%)
D
Downregulated sigDE Trf/+ vs +/+ (455)
Upregulated sigDE Trf/+ vs +/+ (1349)
Class-B ARF DAPseq Targets (4654)
E
30min
Relative quantity
Genotype +/+ +/+ Trf/+ Trf/+
Treatment -IAA +IAA -IAA +IAA
F
+/+ (120)
Trf/+ (1172)
G
Downregulated sigDE Trf/+ IAA vs mock (831)
Upregulated sigDE Trf/+ IAA vs mock (299)
Class-B ARF DAPseq Targets (4654)
H
Genotype +/+ Trf/+
Treatment -IAA +IAA -IAA +IAA
I
ZmARF28-GFP
vector N Trf
MG132 - + - +
ZmARF28 -GFP
αGFP
~58KDa
Ponceau

Fig. S2. *Truffula* mutants do not show changes in *ZmARF28* expression, have defects in auxin signalling transcriptional responses, and the *Trf* mutation results in accumulation of ZmARF28-eGFP in maize protoplasts

(**A-B**) FPKM plots of specific genes in an RNAseq experiment of the vegetative shoot apices of heterozygous *Truffula* (*Trf/+,* orange) and normal (+/+, blue) siblings, dots represent the FPKM value in each library (n=3). (**A**) Expression of genes in the interval that have >5 counts in >=2 samples. (**B**) Expression of *ZmACTIN1* (Zm00001eb348450), *ZmARF28* (Zm00001e408800), and *ZmKNOTTED1* (Zm00001eb055920) (**C**) GO term enrichment analysis of significantly differentially expressed genes (padj<0.05) between *Trf*/+ and +/+ vegetative shoot apices. (**D**) Venn diagram illustrating the overlap between the genes that are significantly downregulated (purple, padj<0.05, Log2FoldChange <-0.5), or significantly upregulated (green, padj<0.05, Log2FoldChange >0.5) in *Trf* compared to normal siblings, with potential class-B ARF regulatory targets (orange, target list is based on DAPseq peak data within 1kb of the gene transcriptional start site for ZmARF13, ZmARF25, ZmARF10 from Galli, et. al., 2018[24]). (**E**) qPCR expression of ZmSAUR27 (Zm00001eb104110), relative to ZmGAPDH (Zm00001eb173410) at 30 minutes with (+IAA) or without (-IAA) IAA treatment in *Trf* (orange) and wildtype (blue) siblings, "*" P-Value<0.05, (students t-test, biological replicates = 3, technical replicates = 3). (**F**) Venn diagram showing overlap of the significantly differentially expressed genes (padj<0.05) in *Trf* (orange) or normal (blue) sibling seedlings treated with auxin (IAA). (**G**) Venn diagram illustrating the overlap between the genes that are significantly downregulated (purple, padj<0.05, Log2FoldChange <-0.5), or significantly upregulated (green, padj<0.05, Log2FoldChange >0.5) in *Trf* treated with 30minutes IAA versus mock treatment with potential class-B ARF regulatory targets (orange). (**H**) Plot of the average FPKM values of all genes expressed in both normal and *Trf* siblings treated with auxin with GO terms associated with auxin signaling and response, colours indicate relative FPKM along the row (blue is lowest, yellow highest), note that significantly differentially expressed genes are not indicated in this plot. (**I**) Representative anti-GFP western blot of ZmARF28-GFP (both wildtype (N) and *Trf* versions) transiently expressed in maize protoplasts, with and without MG132 treatment (n= 3).

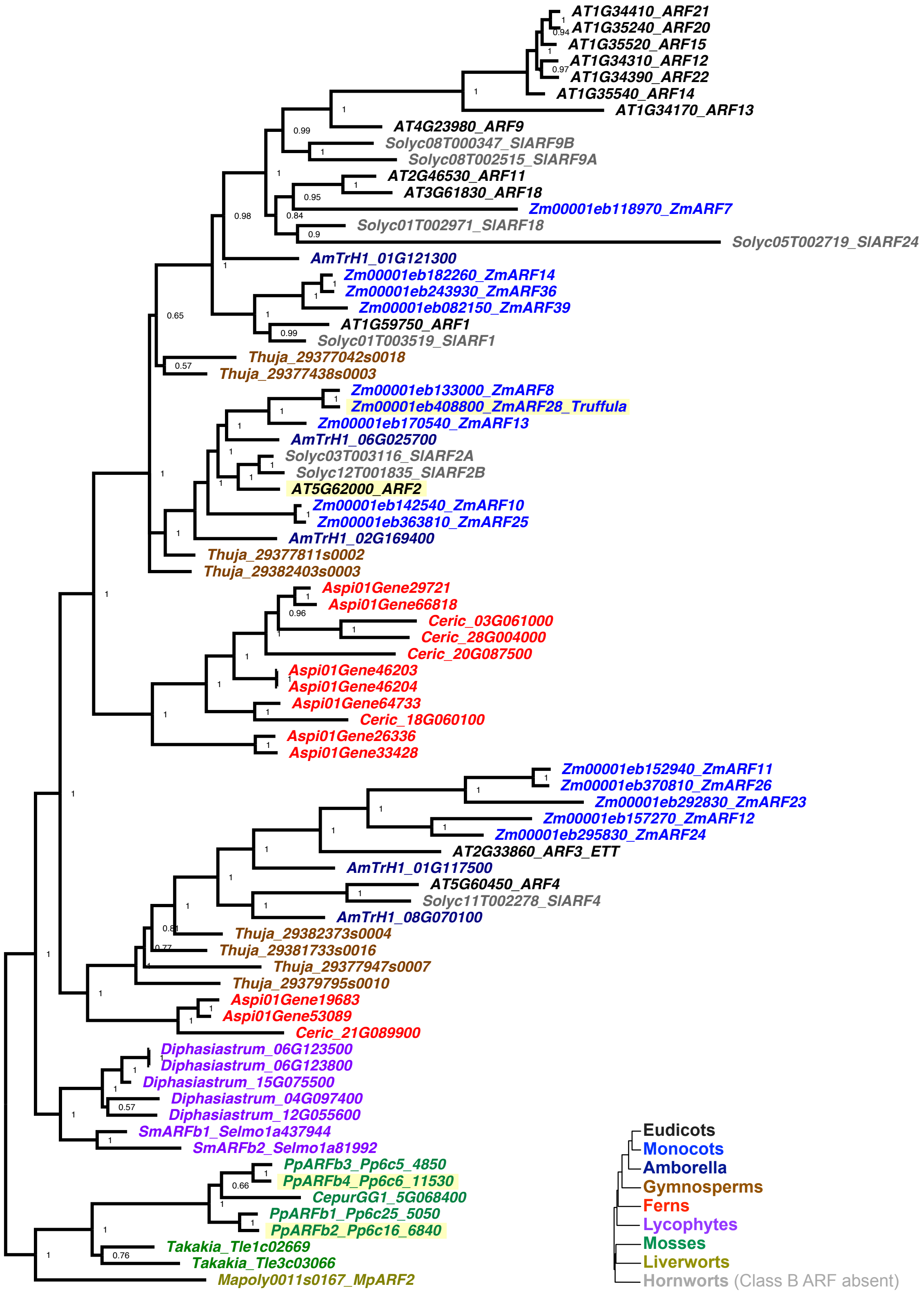

Fig. S3. Bayesian-inferred phylogenetic tree of class-B ARFs from thirteen land plant species

Proteins are colored according to plant clades illustrated in the inset tree showing their relationships. The ZmARF28, AtARF2, PpARFb2, and PpARFb4 sequences are highlighted in yellow. Node labels indicate posterior probabilities supporting the enclosed clades.

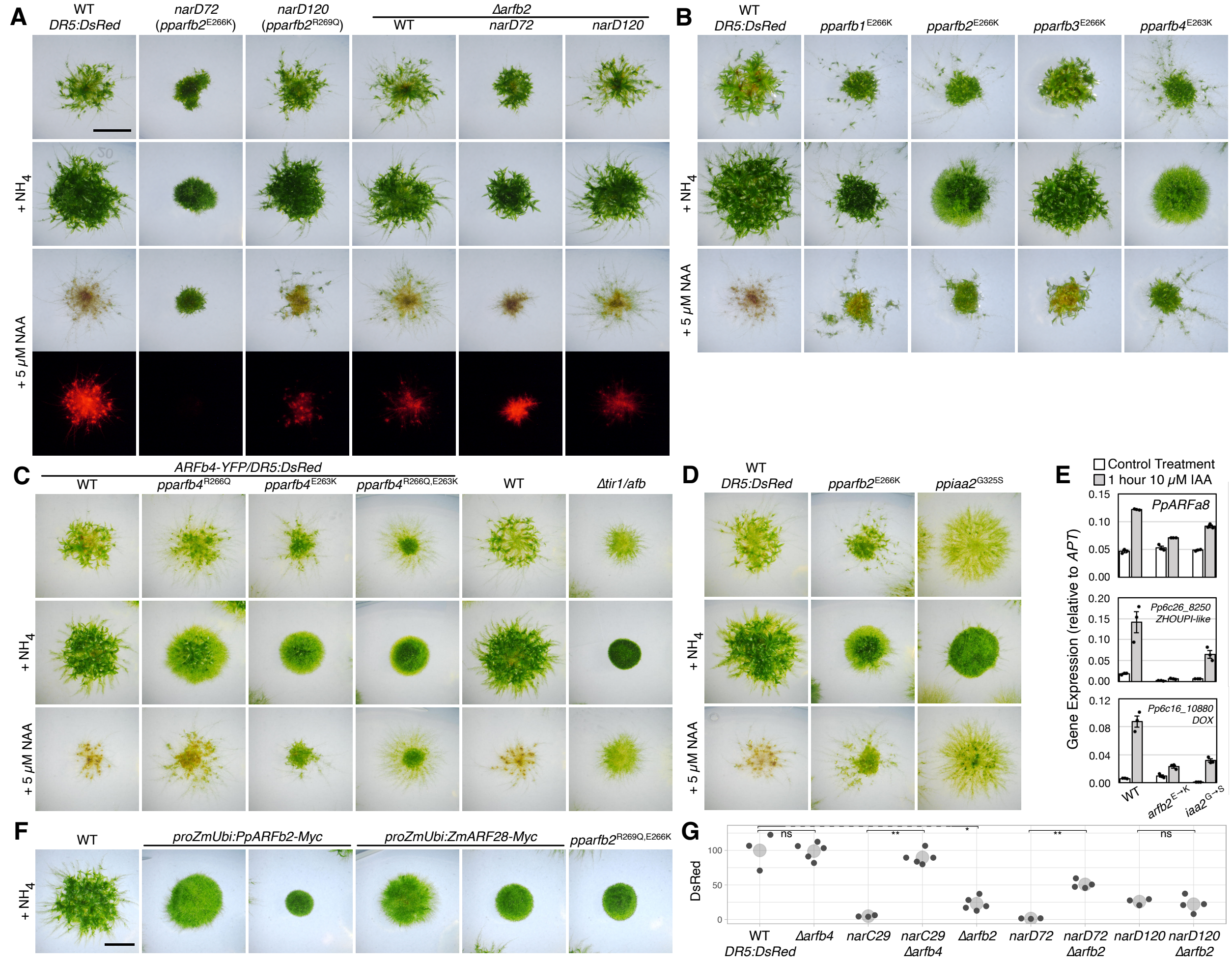

Fig. S4. Mutations in *PpARFb* genes cause auxin resistance

(**A**) Comparison of wild type, *nar* mutants, and Δ*pparfb2* deletion mutants after 21 days of growth on standard medium (BCD) and BCD supplemented with 5 mM ammonium tartrate or 5 µM NAA. Plants grown on NAA were imaged for RFP fluorescence from the *proDR5:DsRed* auxin-response reporter. Leafy shoots in *narD120* are no longer present after deleting the mutant *pparfb2* gene. Scale bar is 5 mm. (**B**) Phenotypes of 21-day-old wild type and gene-edited mutants with E-to-K substitutions in all four class B ARFs showing leafy shoots on NAA media. (**C**) 21-day phenotypes as in panel (A) of the endogenously YFP-tagged *PpARFb4* locus with either the R266Q, E263K, and both gene edits. Δ*tir1/afb* quadruple mutant is shown for comparison. (**D–E**) Auxin-induced gene expression in wild type, $pparfb2^{E266K}$, and $ppiaa2^{G325S}$ (a partially stabilised Aux/IAA degron allele). (**D**) 21-day phenotypes of the strains used in gene expression assay. (**E**) Gene expression of the class-A ARF *PpARFa8* gene, a *ZHOUPI-like bHLH* gene, and a member of the iron/ascorbate-dependent oxidoreductase family. Tissues were treated with 10µM IAA for 1 hour in triplicate before before RNA isolation and RT-PCR. (**F**) Comparison of lines over-expressing the *PpARFb2* and *ZmARF28* genes with the maize *Ubiquitin* promoter to wild type and $pparfb2^{R269Q,E266K}$ lines. Lines with intermediate and strong phenotypes are shown for both. Scale bar is 5 mm. (**G**) Quantification of DsRed signal in NAA-grown WT, *nar* mutants, and deletion lines, normalised to WT. Black dots: individual

plants. Grey circles: mean. n=3 for WT and *nar* mutants, n=2-3 for 2 deletion lines in each background. “**” P-Value<0.01, “*” P-Value <0.05, “ns” not significant, Anova.

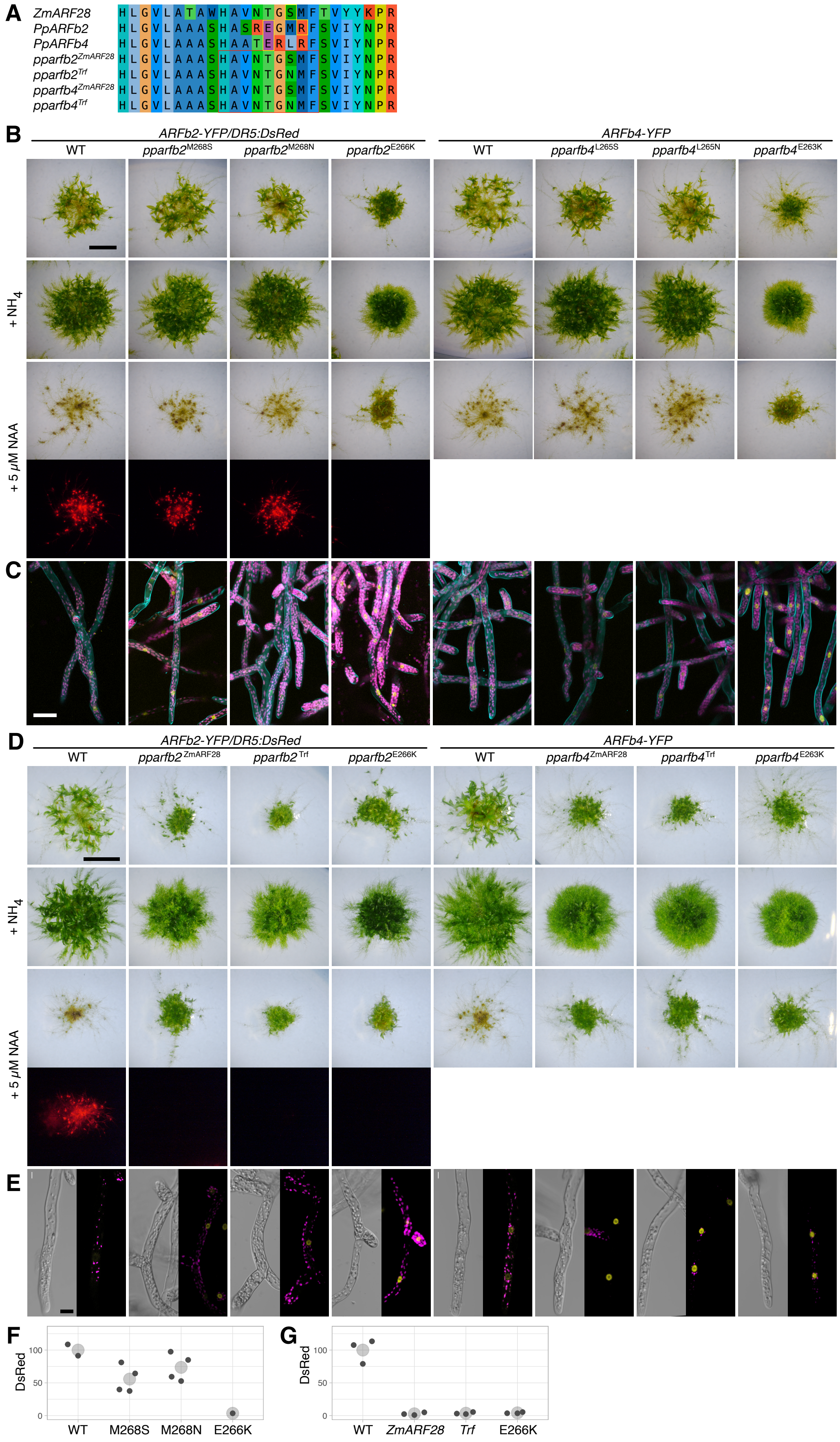

A
ZmARF28
PpARFb2
PpARFb4
pparfb2ZmARF28
pparfb2Trf
pparfb4ZmARF28
pparfb4Trf
B
ARFb2-YFP/DR5:DsRed
ARFb4-YFP
WT
pparfb2M268S
pparfb2M268N
pparfb2E266K
pparfb4L265S
pparfb4L265N
pparfb4E263K
+ NH4
+ 5 μM NAA
C
D
pparfb2ZmARF28
pparfb2Trf
pparfb4ZmARF28
pparfb4Trf
E
F
G
DsRed
100
50
0
M268S
M268N
E266K
ZmARF28
Trf

Fig. S5. *ZmARf28*-like and *Trf*-like substitutions in *PpARFb2 and PpARFb4*

(**A**) Alignment of ZmARF28, PpARFb2, PpARFb4, and the loop swaps introduced into the endogenous loci. Mutations are outlined in yellow and the loop-swap sequences matching ZmARF28 and *Trf* are outlined in red. (**B–C**) Plants with single-amino-acid substitutions in PpARFb2 and PpARFb4 to match either ZmARF28 or *Trf* were indistinguishable from WT and distinct from E-to-K substitutions based on growth assays in BCD, BCDAT, and BCD+5µM NAA media (B) or YFP signal (C). The 4th row of panel (B) show *DR5:DsRed2* reporter expression after growth on NAA. (D-E) Plants with 9-amino-acid swaps of *ZmARF28*-like or *Trf*-like loop sequences into the *PpARFb2* and *PpARFb4* genes were grown as described above. Both swaps result in *nar* phenotypes (D) and increased YFP signal. n≥4. Scalebars: 5mm (B&D), 50µm (D), 20µm (E). (**F-G**) Quantification of DsRed signal in NAA-grown ARFb2-YFP and ARFb2-edited lines shown in (B) and (D) respectively, normalised to WT. Dots: independent edited lines for M268S and M268N or replicates of the same confirmed line for others. Grey circles: mean.

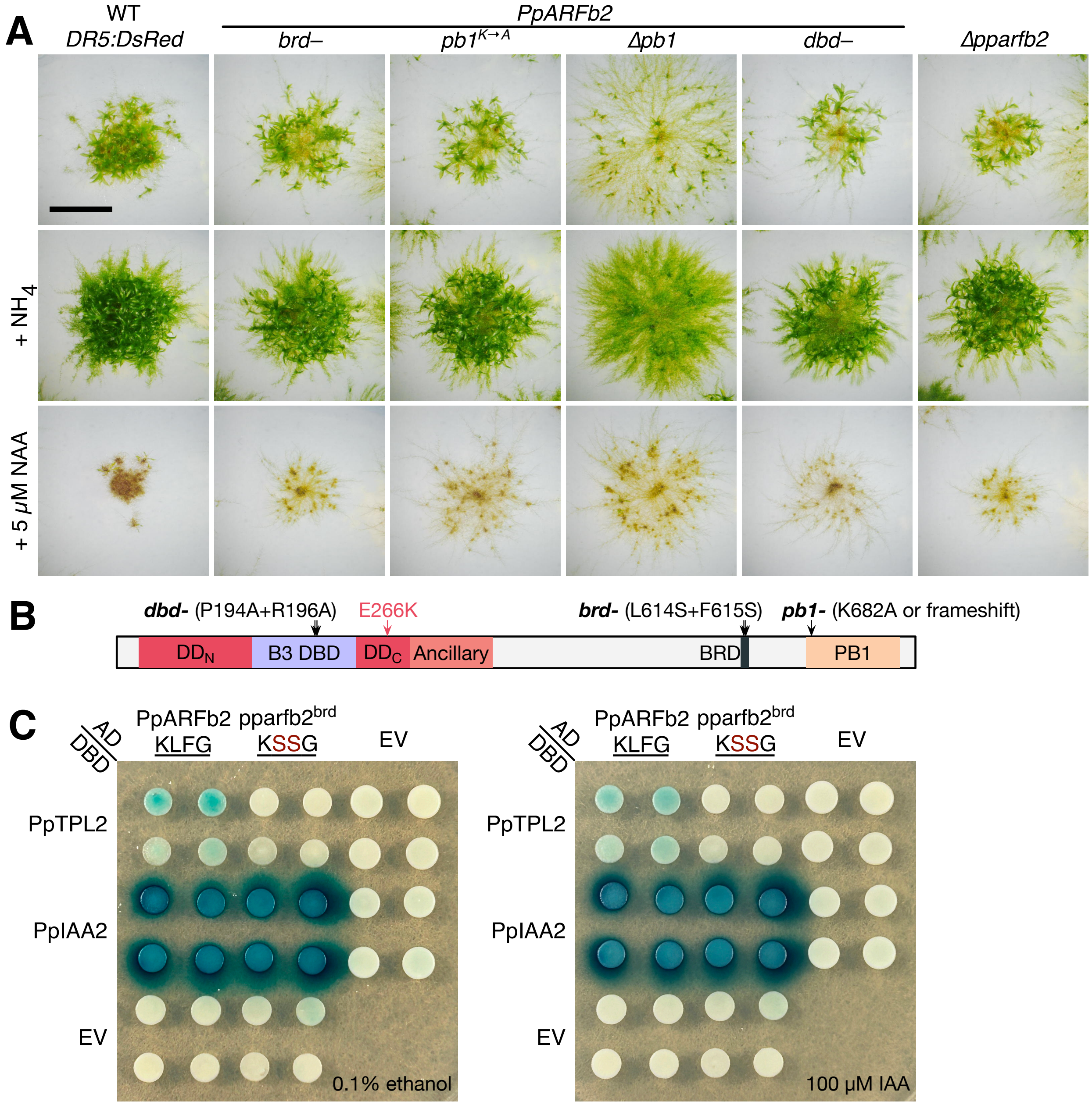

Fig. S6. Mutations in *PpARFb* functional domains

(**A–B**) (**A**) Mutations affecting BRD, PB1, and DBD functions of PpARFb2 have little effect on auxin response, but those with the frameshift mutation at the start of the PB1-encoding region exhibit an increased in protonemal spreading which may indicate auxin hypersensitivity. (**B**) Diagram illustrating the positions of the mutations in panel (**A**) and Fig 3D. (**C**) Yeast-two-hybrid assay showing that wild-type PpARFb2 interacts with TOPLESS homolog PpTPL2 and that this interaction is abolished in the L614S+F615S *brd–* mutant. Both versions interact with the positive control Aux/IAA PpIAA2. The assay was done with and without auxin (IAA).n≥4.

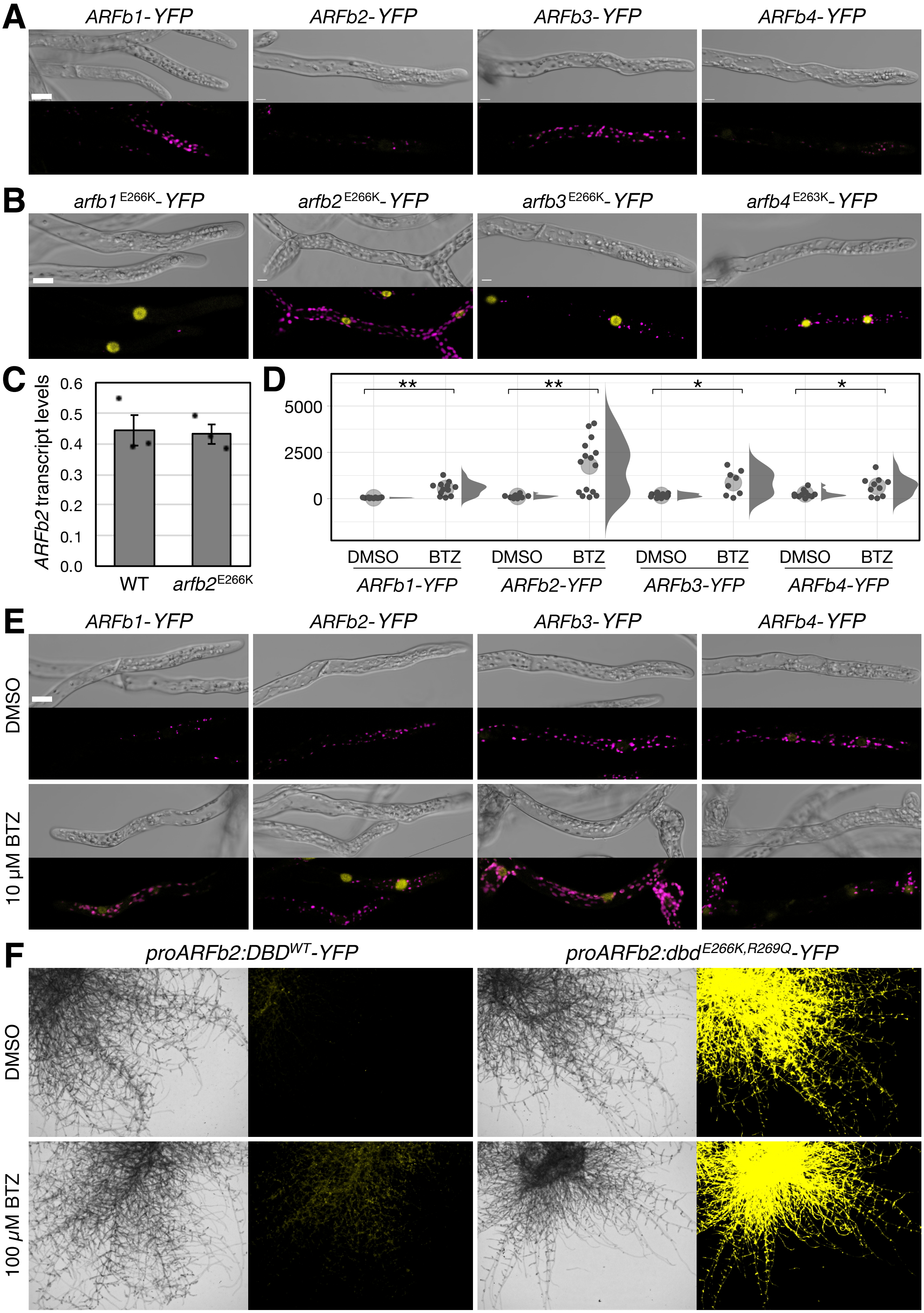
A
ARFb1-YFP
ARFb2-YFP
ARFb3-YFP
ARFb4-YFP
B
arfb1 E266K-YFP
arfb2 E266K-YFP
arfb3 E266K-YFP
arfb4 E263K-YFP
C
ARFb2 transcript levels
0.6
0.5
0.4
0.3
0.2
0.1
0.0
WT
arfb2 E266K
D
**
**
*
*
5000
2500
0
DMSO
BTZ
ARFb1-YFP
ARFb2-YFP
ARFb3-YFP
ARFb4-YFP
E
DMSO
10 μM BTZ
F
proARFb2:DBD WT-YFP
proARFb2:dbd E266K,R269Q-YFP
100 μM BTZ

Fig. S7. Substitutions in *PpARFb* increase protein stability

(**A–B**) YFP fluorescence from wild-type and gene-edited YFP-tagged *PpARFb* loci. DIC images and a merged micrograph for the YFP channel (yellow) and chlorophyll autofluorescence (magenta). The 514 nm excitation laser power was reduced four-fold for acquiring YFP signal from gene-edited lines in panel (C) compared to wild-type loci in panel (B). Scale bars are 10 µm. (**A**) YFP signal is very low or absent for tagged wild-type PpARFb-YFP proteins. (**B**) E-to-K substitutions in all four PpARFb-YFP lines increased YFP fluorescence dramatically. (**C**) *PpARFb2* transcript levels are not significantly altered by the E266K mutation in tissue grown for 1 week on BCDAT medium. Transcript levels from triplicate samples are normalized to that of the *APT* gene. (**D–E**) Overnight bortezomib (BTZ) treatment increased the YFP signal in fusions to all four *ARFb* genes relative to controls (DMSO). (**D**) Quantification of YFP signal in control-treated and in BTZ-treated lines. "**" P-Value <0.01, "*" P-Value < 0.05, t-test. (**E**) Imaging as in panels B and C. (**F**) The transgenic lines used for immunoprecipitation. YFP signal for ARFb2's DBD with the wild-type loop domain is only broadly detectable after bortezomib treatment (16h), while that for the E266K+R269Q mutant version accumulates even without treatment. Both transgenes were expressed using the *ARFb2* promoter, had P194A+R196A substitutions (*dbd–*), and had silent mutations in the *miR1219* target. n≥4.

Table S1. SSR Primers used in mapping of the *Trf* mutation

Reference genomes used: Zm-B73-REFERENCE-NAM-5.0, Mo17 CAU Assembly (Zm00014a), B73 RefGen_v3 (MGSC), W22 NRGene 2.0 assembly. (see excel spreadsheet)

Table S2. Genes annotated in the *Trf* mapping interval as determined by SSR primer mapping in the 5b+, NAM5.0, W22-2.0, and Mo17-CAU1.0 reference genomes available on maize GDB (Up to date for March 2023 versions)
(see excel spreadsheet)

Table S3. Significant and moderate effect SNP variants identified in the *Trf* mapping interval using SnpEff.

NAM5.0 Gene ID	SNP Variant	Predicted amino acid change	Notes
Zm00001eb408750	chr10: 13,501,412 C>T	Arg331Lys in T001	natural variant in Mo17
Zm00001eb408750	chr10: 13,501,725 G>T	Leu227Met in T001	natural variant in Mo17
Zm00001eb408750	chr10: 13,501,946 C>T	Cys153Tyr in T001	natural variant in Mo17
Zm00001eb408750	chr10: 13,503,214 C>A	Ala62Ser in T001	natural variant in Mo17 and W22
Zm00001eb408800	chr10: 13,676,774 G>A	T004 Ser281Asn, T005 Ser280Asn, T002 Ser281Asn, T006 Ser281Asn, T001 Ser281Asn	conserved serine across B73, W22, Mo17
Zm00001eb408800	chr10: 13,678,064 G>A	T003 Thre558IIe, T005 Thr558Ile, T002 Thr559IIe, T004 Thr559Ile, T006 Thr559Ile, T001 Thr559Ile	Variable residue across B73, W22, Mo17
Zm00001eb408820	chr10: 13,953,240 C>A	T001 Asp99Glu	Gene annotation absent from Mo17 and W22 reference genomes, CML52 has Glu at this residue.

Table S4. All significantly differentially expressed genes (padj<0.05) in the RNAseq comparison of *Trf* vs normal sibling vegetative shoot apices.
(see excel spreadsheet)

Table S5. All significantly enriched GO terms in the RNAseq comparison of *Trf* vs normal sibling vegetative shoot apices.
(see excel spreadsheet)

Table S6. All significantly differentially expressed genes (padj<0.05) in the RNAseq comparison of normal siblings +IAA vs normal sibling +mock treatment vegetative shoot apices.
(see excel spreadsheet)

Table S7. All significantly differentially expressed genes (padj<0.05) in the RNAseq comparison of *Trf* siblings +IAA vs *Trf* sibling +mock treatment vegetative shoot apices.
(see excel spreadsheet)

Table S8. PpARFb2 Loop-Region Indels

Line	Sequence(aa 261-269)	Phenotype
WT	**SHASREGMR**	WT
T20-2 (E266K)	**SHASRKGMR**	intermediate
T20-4	**SHAP-EGMR**	strong
T23-2	**SHAAQSRSTKGMR**	strong
T21-3	**SHAS-EGMR**	strong
T21-4	**SHAC-EGMR**	strong
T21-2	**SHA-FMCMR**	intermediate
T23-1	**SH---EGMR**	intermediate
T21-1	**SHDA-EGMR**	intermediate

Table S9. Primers used in moss cloning
(see excel spreadsheet)

Table S10. AlphaFold pTM values for the homology models presented in Fig.2

DBD	pTM
**ZmARF28**	0.81
**trf**	0.81
**PpARFb2**	0.85
**Pparfb2E266K**	0.85
**Pparfb2R269K**	0.85
**PpARFb4**	0.85
**Pparfb4E263K**	0.86

Maize Protoplast ZmARF28-GFP expression western blot (Fig.S2I)

Matched images of the blot. (A) automatic tiff multichannel overlay image from the Azure 300 imager. (B) only the greyscale channel from the Azure 300 imager. (C) Ponceau staining of the same blot. ZmARF28-GFP is ~135kDa, the red ladder marker is 75KDa, the next blue marker is 100, and the next up is 135KDa. .

2 replicates tested, samples arranged as:
Ladder/ ZmARF28-N-GFP - MG132 / ZmARF28-N-GFP + MG132 / ZmARF28-Trf-GFP - MG132 / ZmARF28-Trf-GFP + MG132/
ZmARF28-N-GFP - MG132 / ZmARF28-N-GFP + MG132 / ZmARF28-Trf-GFP - MG132 / ZmARF28-Trf-GFP + MG132

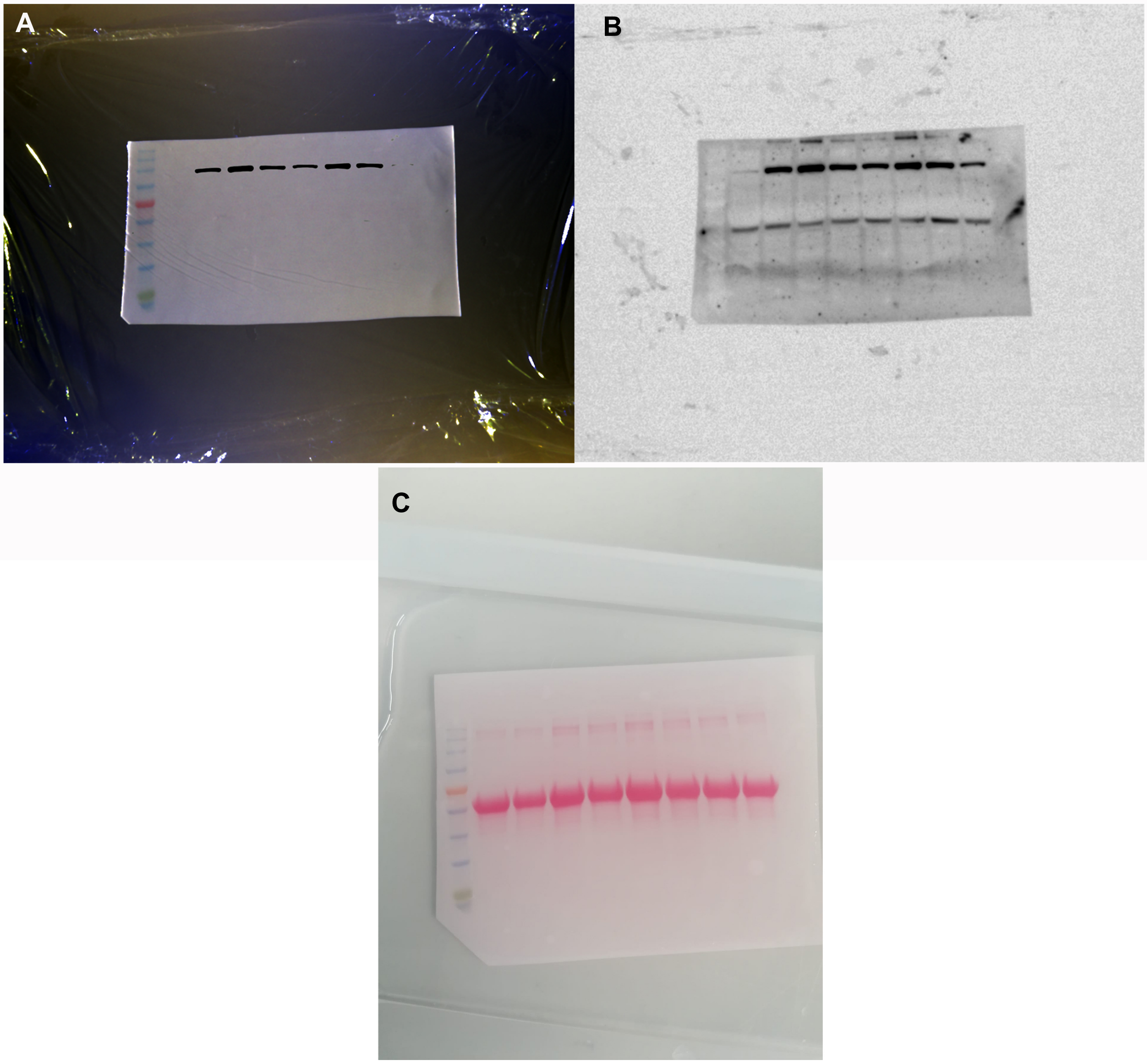

Supplement: Supplementary file 1 — Supplementary Figs. 1–7, legends for Supplementary Tables 1–10 and unprocessed western blots for Supplementary Fig. 2I. [file 41477_2025_1973_MOESM1_ESM.pdf]
