# Supplementary material for: Comparative mutant analyses reveal a novel mechanism of ARF regulation in land plants
Source: Nat Plants. 2025 Apr 11;11(4):821–35. doi: 10.1038/s41477-025-01973-3 (PMC12014491; doi:10.1038/s41477-025-01973-3)
Supplement: Supplementary file 2 — Reporting Summary [file 41477_2025_1973_MOESM2_ESM.pdf]

nature portfolio

Corresponding author(s): *Dr Annis Richardson (annis.richardson@ed.ac.uk) Prof. Mark Estelle Prof. Lucia Strader

Last updated by author(s): 2025/01/21

Reporting Summary

Nature Portfolio wishes to improve the reproducibility of the work that we publish. This form provides structure for consistency and transparency in reporting. For further information on Nature Portfolio policies, see our Editorial Policies and the Editorial Policy Checklist.

Statistics

For all statistical analyses, confirm that the following items are present in the figure legend, table legend, main text, or Methods section.

n/a	Confirmed	
☐	☒	The exact sample size (*n*) for each experimental group/condition, given as a discrete number and unit of measurement
☐	☒	A statement on whether measurements were taken from distinct samples or whether the same sample was measured repeatedly
☐	☒	The statistical test(s) used AND whether they are one- or two-sided<br>*Only common tests should be described solely by name; describe more complex techniques in the Methods section.*
☐	☒	A description of all covariates tested
☐	☒	A description of any assumptions or corrections, such as tests of normality and adjustment for multiple comparisons
☐	☒	A full description of the statistical parameters including central tendency (e.g. means) or other basic estimates (e.g. regression coefficient) AND variation (e.g. standard deviation) or associated estimates of uncertainty (e.g. confidence intervals)
☐	☒	For null hypothesis testing, the test statistic (e.g. *F*, *t*, *r*) with confidence intervals, effect sizes, degrees of freedom and *P* value noted<br>*Give P values as exact values whenever suitable.*
☐	☒	For Bayesian analysis, information on the choice of priors and Markov chain Monte Carlo settings
☐	☒	For hierarchical and complex designs, identification of the appropriate level for tests and full reporting of outcomes
☒	☐	Estimates of effect sizes (e.g. Cohen's *d*, Pearson's *r*), indicating how they were calculated

Our web collection on statistics for biologists contains articles on many of the points above.

Software and code

Policy information about availability of computer code

Data collection	*Provide a description of all commercial, open source and custom code used to collect the data in this study, specifying the version used OR state that no software was used.*
Data analysis	All software and code used in the analysis is provided with relevant references in the manuscript materials and methods. All code used for the maize genomic and transcriptomic analyses is available via the gitub page provided in the acknowledgments and data availability statement. (https://github.com/ThePlantShapeLab/Truffula) . For WGS data: Sequence data was downloaded via ftp from Novogene servers and passed through the following analysis pipeline: FASTPQ> Bowtie2 alignment to the B73_Reference_Nam5.0 genome > samtools mpileup variant calling > SNPeff and variant filtering in R. For RNAseq data: Sequence data was downloaded via ftp from Novogene servers and passed through the following analysis pipeline: FASTPQ > HiSAT2 alignment to the Zea mays Reference NAM5.0 genome (https://www.maizegdb.org/genome/assembly/Zm-B73-REFERENCE-NAM-5.0) > FeatureCounts > DeSeq2. GO term analysis was carried out using the GAMER Maize annotations (accessed via https://datacommons.cyverse.org/browse/iplant/home/shared/commons_repo/curated/Carolyn_Lawrence_Dill_GOMAP_Maize_MaizeGDB_B73_NAM_5.0_December_2021.r1/3_final-result/3.1_B73v5.MaizeGDB.CLEANED.gaf.gz) and the GOSeq R package. All software was installed using command line during 2022, and genome data was accessed and downloaded in 2022. Maize DAPseq data published in Galli, et. al. 2018 is available here https://data.waksman.rutgers.edu/aj2/gallavotti/ZmARFs

For manuscripts utilizing custom algorithms or software that are central to the research but not yet described in published literature, software must be made available to editors and reviewers. We strongly encourage code deposition in a community repository (e.g. GitHub). See the Nature Portfolio guidelines for submitting code & software for further information.

Data

Policy information about availability of data

All manuscripts must include a data availability statement. This statement should provide the following information, where applicable:

- Accession codes, unique identifiers, or web links for publicly available datasets
- A description of any restrictions on data availability
- For clinical datasets or third party data, please ensure that the statement adheres to our policy

Data and materials availability: All original numerical, sequence and image data is included in the manuscript or freely available via The Plant Shape Lab collection on the University of Edinburgh DataShare service (https://datashare.ed.ac.uk/handle/10283/8938), mutant and parental P. patens raw sequence reads are available at BioProject (PRJNA1210789). Seeds and plant material are available on request from Richardson (maize), Estelle (P.patens), Strader (Arabidopsis).
Maize B73 NAM5.0 genome was accessed here: https://www.maizegdb.org/genome/assembly/Zm-B73-REFERENCE-NAM-5.0, Maize GO annotations were accessed here: https://datacommons.cyverse.org/browse/iplant/home/shared/commons_repo/curated/Carolyn_Lawrence_Dill_GOMAP_Maize_MaizeGDB_B73_NAM_5.0_December_2021.r1/3_final-result/3.1_B73v5.MaizeGDB.CLEANED.gaf.gz, Maize DAPseq data published in Galli, et. al. 2018 is available here https://data.waksman.rutgers.edu/aj2/gallavotti/ZmARFs

Research involving human participants, their data, or biological material

Policy information about studies with human participants or human data. See also policy information about sex, gender (identity/presentation), and sexual orientation and race, ethnicity and racism.

Reporting on sex and gender	N/A
Reporting on race, ethnicity, or other socially relevant groupings	N/A
Population characteristics	N/A
Recruitment	N/A
Ethics oversight	N/A

Note that full information on the approval of the study protocol must also be provided in the manuscript.

Field-specific reporting

Please select the one below that is the best fit for your research. If you are not sure, read the appropriate sections before making your selection.

☒ Life sciences ☐ Behavioural & social sciences ☐ Ecological, evolutionary & environmental sciences

For a reference copy of the document with all sections, see nature.com/documents/nr-reporting-summary-flat.pdf

Life sciences study design

All studies must disclose on these points even when the disclosure is negative.

Sample size	Appropriate samples sizes were determined based on methodology established in the published literature. In all experiments at least 3 biological replicates were used, and 2-3 technical replicates.
Data exclusions	For transcriptomic data samples that failed quality checks carried out by the sequencing company were rejected. For sequenced libraries PCA anlayses was used to assess if the libraries clustered before continuing with analyses.
Replication	At least 2 technical replicates were carried out for all experiments, with at least 3 biological replicates per technical replicate. All replicates showed results consistent with each other.
Randomization	Samples were genotyped to confirm mutant/ normal then randomly assigned pools where relevant.
Blinding	N/A Blinding was not use din this study, and human bias was not deemed to be of high risk, as all experimental work was analytical and did not rely upon human interpretation to define the result.

Reporting for specific materials, systems and methods

We require information from authors about some types of materials, experimental systems and methods used in many studies. Here, indicate whether each material, system or method listed is relevant to your study. If you are not sure if a list item applies to your research, read the appropriate section before selecting a response.

Materials & experimental systems

n/a	Involved in the study
☐	☒ Antibodies
☒	☐ Eukaryotic cell lines
☒	☐ Palaeontology and archaeology
☒	☐ Animals and other organisms
☒	☐ Clinical data
☒	☐ Dual use research of concern
☐	☒ Plants

Methods

n/a	Involved in the study
☒	☐ ChIP-seq
☒	☐ Flow cytometry
☒	☐ MRI-based neuroimaging

Antibodies

Antibodies used	Custom antibodies to ZmARF28 were generated in guineapig by Author Dr Jazmin Abraham-Juarez. Commeracially available antibodies used were:<br>mouse anti-GFP (Roche #11814460001)<br>anti-mouse-HRP (Cell Signaling Technology, #7076)<br>Anti-guinea pig-HRP secondary antibody (Thermo Fisher Scientific catalog no. A18769)<br>rabbit anti-GFP polyclonal antibody (Invitrogen, A11122)<br>P4D1 mouse monoclonal antibody (Cell Signaling Tech, 3936T)<br>goat-anti-rabbit (Millipore, A6154)<br>goat-anti-mouse (Bio-Rad, 1706516)
Validation	Custom antibodies were validated using western blot, and indicated single bands of the correct size for ZmARF28 from crude plant protein extract (as in Figure 4). Commercial antibodies were validated by the manufacturers for cross-reactivity using western blots and ELISA assays. In the lab we tested for specificity using western blot before carrying out the final data-collection experiments, looking for signal intensity and specificity when compared to control purified protein samples.

Dual use research of concern

Policy information about dual use research of concern

Hazards

Could the accidental, deliberate or reckless misuse of agents or technologies generated in the work, or the application of information presented in the manuscript, pose a threat to:

No	Yes
☒	☐ Public health
☒	☐ National security
☒	☐ Crops and/or livestock
☒	☐ Ecosystems
☒	☐ Any other significant area

Experiments of concern

Does the work involve any of these experiments of concern:

No	Yes
☒	☐ Demonstrate how to render a vaccine ineffective
☒	☐ Confer resistance to therapeutically useful antibiotics or antiviral agents
☒	☐ Enhance the virulence of a pathogen or render a nonpathogen virulent
☒	☐ Increase transmissibility of a pathogen
☒	☐ Alter the host range of a pathogen
☒	☐ Enable evasion of diagnostic/detection modalities
☒	☐ Enable the weaponization of a biological agent or toxin
☒	☐ Any other potentially harmful combination of experiments and agents

Plants

Seed stocks	We thank Gerry Neuffer for the kind donation of the original Trf maize seed, generated by GN in an EMS screen. W22 and B73 maize seed was provided by the Hake/ Richardson lab stocks. Moss mutants were generated in the Estelle lab/
Novel plant genotypes	We thank Gerry Neuffer for the kind donation of the original Trf maize seed, generated by GN in an EMS screen. Moss mutants were generated via irradiation with 150 mJ/cm2 of UV light , and via CRISPR editing.
Authentication	All plant lines were genotyped using PCR and subsequence sanger sequencing. The maize Trf mutant line was additionally subject to whole-genome sequencing to confirm mapping interval.
