# Supplementary material for: Comparative mutant analyses reveal a novel mechanism of ARF regulation in land plants
Source: Nat Plants. 2025 Apr 11;11(4):821–35. doi: 10.1038/s41477-025-01973-3 (PMC12014491; doi:10.1038/s41477-025-01973-3)
Supplement: Supplementary file 4 — Unprocessed western blot images for Fig. 4c,e,g. [file 41477_2025_1973_MOESM4_ESM.pdf]

Western blot of ZmARF28 protein accumulation in maize (Fig.4C)

Matched images of the blot from Fig.4C.
(A) developed X-ray film of anti-ARF28 western blot. (B) developed X-ray film of anti-TUBULIN western blot.
2 biological replicates for each genotype.
ZmARF28-GFP is ~135kDa, the red ladder marker is 75KDa, the next blue marker is 100, and the next up is 135KDa.

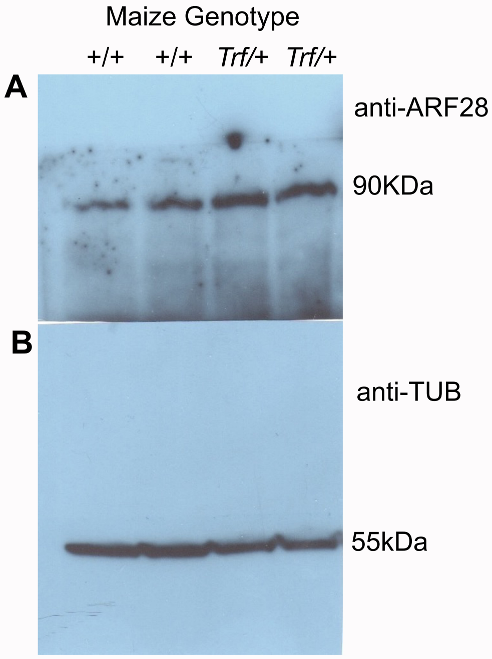

Western blot of *ZmARF28-GFP* expression in *N.benthamiana* (Fig.4E)

Matched images of the blot from Fig.4E.
(A) Greyscale channel from the Azure 300 imager of the anti-GFP western blot. (B) Ponceau staining of the same blot.
ZmARF28-GFP is ~135kDa, the red ladder marker is 75KDa, the next blue marker is 100, and the next up is 135KDa.

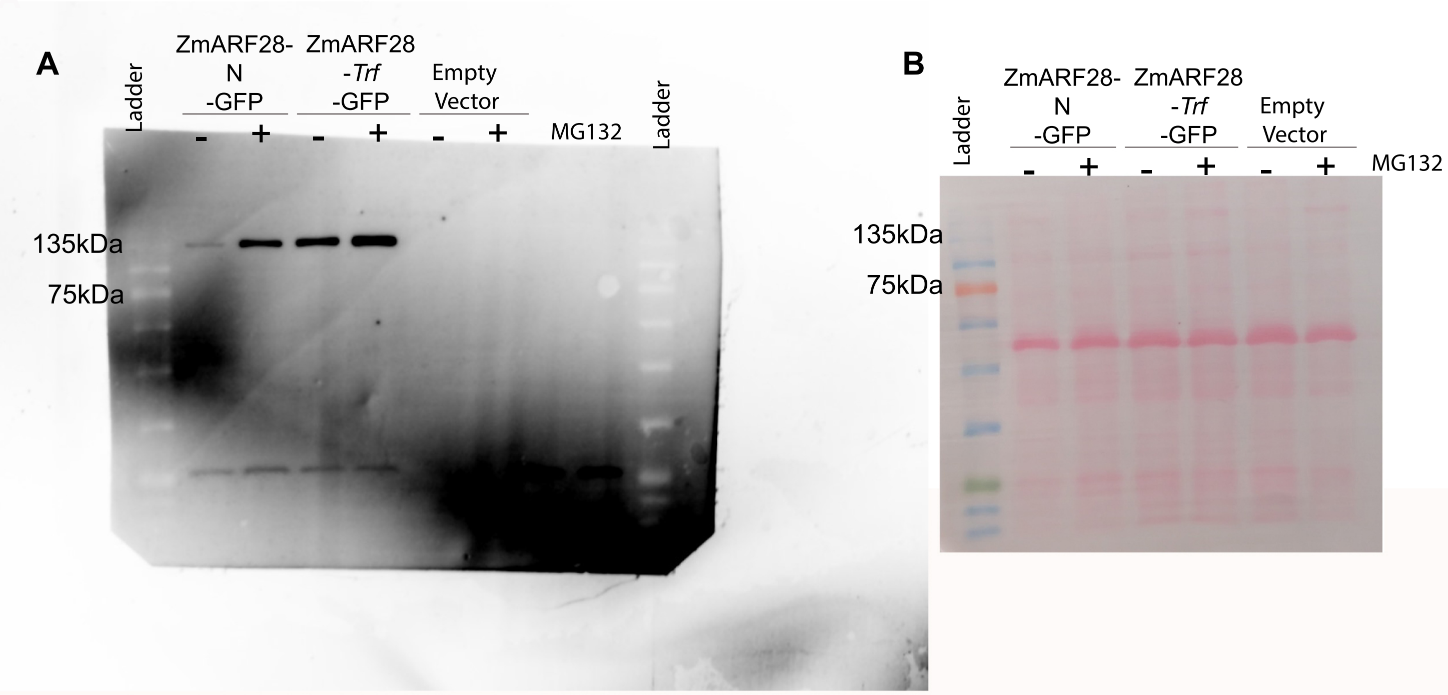

Western blots of *PpARF2-DBD-YFP* (Fig.4G)

Matched images of the blots in Fig.4G.
Input samples with matched gels for anti-GFP (A) and anti-Ubiquitin blots (B) with respective ponceau staining images.
GFP immunoprecipitation samples in matched gels for anti-Ubiquitin (C) and anti-GFP(D) with respective ponceau staining
The ladder shown in the left hand side of the ponceau images is 75KDa at the red band.
3 replicates shown for control (Reute WT), normal (DBD-YFP) and mutant (dbd-YFP) samples.

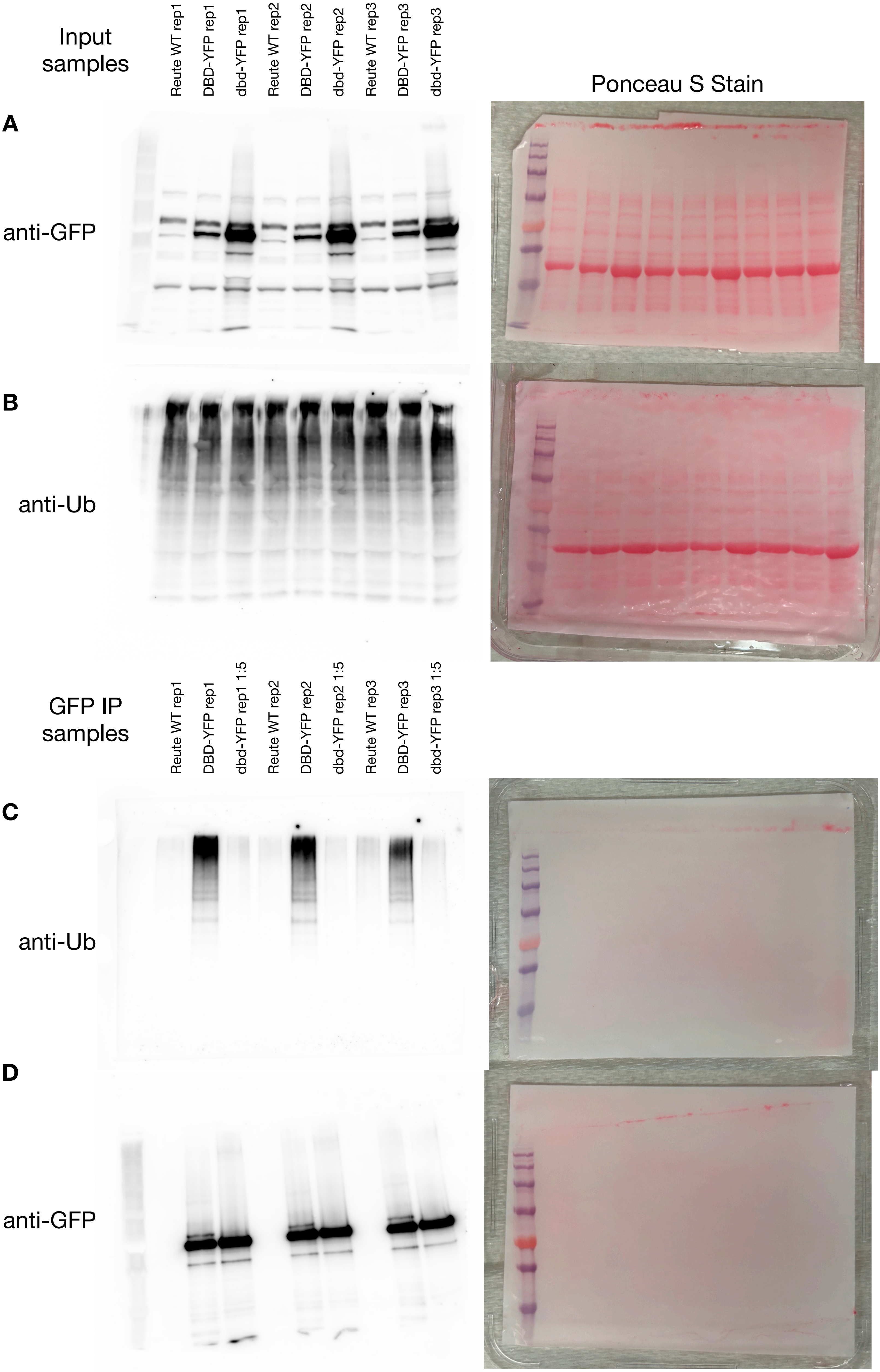
Input samples
Reute WT rep1
DBD-YFP rep1
dbd-YFP rep1
Reute WT rep2
DBD-YFP rep2
dbd-YFP rep2
Reute WT rep3
DBD-YFP rep3
dbd-YFP rep3
Ponceau S Stain
A
anti-GFP
B
anti-Ub
GFP IP samples
Reute WT rep1
DBD-YFP rep1
dbd-YFP rep1 1:5
Reute WT rep2
DBD-YFP rep2
dbd-YFP rep2 1:5
Reute WT rep3
DBD-YFP rep3
dbd-YFP rep3 1:5
C
anti-Ub
D
anti-GFP
